# Phytochemical analysis and neuroprotective potential of *Achillea santolina* L. fractions

**DOI:** 10.1038/s41598-025-98887-z

**Published:** 2025-05-08

**Authors:** Passent M. Abdel-Baki, Nariman E. Mahdy, Rana M. Ibrahim, Shymaa A. El Badawy, Sara E. Ali, Marwa A. Ibrahim, Marwa S. Khattab, Ahmed A. El-Rashedy, Shimaa R. Emam

**Affiliations:** 1 Department of Pharmacognosy, Faculty of Pharmacy, Cairo University, Kasr-El-Ainy Street, Cairo, 11562 Egypt; 2 Department of Pharmacology, Faculty of Veterinary Medicine, Cairo University, Giza, 12211 Egypt; 3 Department of Physiology, Faculty of Veterinary Medicine, Cairo University, Giza, 12211 Egypt; 4 Department of Biochemistry and Molecular Biology, Faculty of Veterinary Medicine, Cairo University, Giza, 12211 Egypt; 5 Department of Pathology, Faculty of Veterinary Medicine, Cairo University, Giza, 12211 Egypt; 6 Chemistry of Natural and Microbial Products Department, National Research Center (NRC), Dokki, Giza 12622 Egypt; 7 Department Organic and Medicinal Chemistry, Faculty of Pharmacy, University of Sadat City, El Sadat, Menoufia 32897 Egypt

**Keywords:** *Achillea santolina*, HPLC, Docking, Inflammation, Monosodium glutamate, ADME, Computational biology and bioinformatics, Plant sciences, Neurology

## Abstract

Phytochemical characterization, and biological assessment of the neuroprotective activities of *Achillea santolina* L. methanolic extract (AS), its methylene chloride fraction (MF), butanol fraction (BF) and their isolated compounds were investigated. Twenty-two compounds were identified and quantified in AS using HPLC. Luteolin and kaempferol were isolated from MF. Isovitexin and kaempferol 3-*O*-glucoside were isolated from BF. The anti-inflammatory activity of the isolated compounds was determined by employing cyclooxygenase-2 (COX-2) and 5-lipoxygenase (5-LOX) inhibitory assays. Neuroprotective activities of AS, MF and BF were investigated against monosodium glutamate (MSG) induced neurotoxicity in rats. Brain damage amended significantly as indicated by the decreased lactate dehydrogenase and tumor necrosis factor. Brain oxidative status was restored as indicated by increased glutathione and decreased lipid peroxidation. AS, MF and BF markedly attenuated histopathological alterations in cerebral cortex and downregulated expression of COX-2, IL-1B, IL-10 genes. The affinity of the isolated compounds for Human Heme Oxygenase-1, 5-LOX, Kelch-like ECH-associated protein and COX-2 was studied. Molecular dynamics simulation and ADME study proved that isovitexin has stable conformations and binding patterns with the active sites of the studied enzymes. For the first time, the neuroprotective potential of AS, fractions and isolated compounds was determined supported by anti-inflammatory study.

**Supplementary Information:**

The online version contains supplementary material available at 10.1038/s41598-025-98887-z.

## Introduction

Monosodium glutamate (MSG) is derived from naturally occurring amino acid L-glutamic acid and used extensively as flavor enhancer in various food industries. The daily consumption of MSG is spreading worldwide to be estimated 3–4 g, however 0.3 g and 1 g is safe^1^. According to various health authorities, including the European Food Safety Authority (EFSA), the Joint FAO/WHO Expert Committee on Food Additives (JECFA) and the Food and Drug Administration (FDA), MSG is usually deemed safe. However, some clinical studies have confronted its safety. Glutamate is a major substrate for energy production in enterocytes, an intermediate substance in protein metabolism, a precursor of considerable metabolites such as glutathione (GSH, oxidative stress modulator) or N-acetylglutamate (metabolic regulator), and an excitatory neurotransmitter of the central nervous system (CNS)^2^. The extracellular accumulation of high concentrations of glutamate leads to excessive stimulation of its receptors. Consequently, its target neurons become damaged. This is regarded as a pathological condition called excitotoxicity, which is linked to neurodegenerative diseases^3,4^.

Moreover, overactivation of glutamate pathways, enhances accumulation of β-amyloid in hippocampus^5^ and decreases γ-aminobutyric acid (GABA)^6^. In addition, chronic neurodegeneration, brain damage, status epilepticus, traumatic injuries and cerebral ischemia can be denoted by the increase in CNS glutamate concentration^7^.

Neurodegenerative diseases are linked to neuroinflammation caused by inflammatory mediators comprising cyclooxygenase-2 (COX-2) derived prostaglandins and 5-lipoxygenase (5-LOX) derived leukotrienes, as well as oxidative stress^8^. Also, neuroinflammation triggers the production of free radicals. As a result, it is recommended to provide the patient with an adequate antioxidant as an additional therapy for neuroinflammation.

Heme oxygenases (HOs) are enzymes that regulate the amount of heme in mammalian bodies. This family of enzymes is comprised of three isoforms which are HO-1, HO-2, and HO-3. Several lines of evidence suggest that HO-1 dysregulation is linked to CNS aging, brain inflammation and neurodegenerative disorders^9^. In human cellular cytoplasmic homeostasis, a transcription factor; nuclear factor erythroid 2-related factor 2 (Nrf2) is responsible for the upregulation of the cell detoxifying enzymes expression due to oxidative stress. Nrf2–Keap1 system, Nrf2 levels controlled by Kelch-like ECH-associated protein 1 (Keap1), is regarded as a promising therapeutic strategy for oxidative and inflammatory stress-related disorders^10^.

Drugs targeting specific targets may not be enough to halt the progression of neurodegenerative diseases due to their complexity. Developing multi-targeted medications that combine several pharmacological activities can lead to more effective therapy.

*Achillea* consists of around 130 flowering species, spread in the Northern hemisphere mainly Europe, Asia, North America and the Middle East^11^. *Achillea i*s regarded as one of the most significant genera of family Asteraceae. The aerial parts of the different species are widely used in traditional medicine. The genus *Achillea* is represented in Egypt by two species; *A. fragrantissima* (Forssk.) Sch. Bip. (common name Qaysoom) and *A. santolina* L. (common name Be’eitheran)^12,13^.

In Bedouin traditional medicine, the aerial parts and roots of *A. santolina* are used for central nervous system disorders, as analgesic and for fits of hysteria^13^. It is also used as anti-inflammatory, carminative, stomachic, antidiabetic and anti-helminthic^14^. *A. santolina* was reported to exhibit antioxidant^15^, antidiabetic^16^, antibacterial^16,17^, analgesic and ani-inflammatory activities^18^. Various active constituents were reported in *A. santolina* including flavonoids, phenolic acids and sesquiterpene lactones^16^ as well as essential oil^17,19^. Despite several ethnopharmacological and phytotherapeutic reports on its anti-inflammatory, antioxidant and neuropharmacological effects, no evidence of its potential as a neuroprotective drug was discovered.

Herbal formulations are increasingly being used to treat neurological problems and have been scientifically validated for their potential to cure neurodegenerative conditions^20^. Accordingly, the aim of this research was to investigate the efficiency of *Achillea santolina* methanolic extract (AS) and its methylene chloride (MF) and butanol (BF) fractions against MSG-induced neurotoxicity and whether it could exert protective effects against neuronal inflammatory response and oxidative stress on* in vitro*, *in vivo* and *in silico *levels. Moreover, our study aimed to identify the potential involvement of phytochemical composition of AS fractions on its mediated neuroprotection.

Qualitative and quantitative analysis of the phenolics of AS was performed using high performance liquid chromatography (HPLC), followed by fractionation of the AS to obtain methylene chloride fraction (MF) and butanol fraction (BF) as well as isolation of their major compounds. Spectrophotometric determination of the total phenolic (TPC) and total flavonoid (TFC) contents were conducted on AS, MF and BF respectively. AS and its fractions [methylene chloride (MF) and butanol (BF)] were evaluated for their *in vitro* antioxidant potential using three assays: 2,2’-azino-bis(3-ethylbenzthiazoline-6-sulfonic acid) (ABTS), ferric reducing antioxidant power (FRAP) and oxygen radical absorbance capacity (ORAC). In addition, the anti-inflammatory [cyclooxygenase-2 (COX-2) and 5-lipoxygenase (5-LOX)] activity of the isolated compounds was determined. Furthermore, molecular docking was utilized to examine the potential binding modes and interactions of the isolated compounds with HO-1, 5-LOX, Keap1 and COX-2 active sites to investigate the inhibitory potentials. Additionally, the physicochemical features of the isolated compounds were estimated by ADME computational parameters simulation. A molecular dynamic simulation was used to expect the activity of the isolated compounds on the enzymes’ active sites, as well as their interaction and stability. These findings propose the multi-targeted neuroprotective activity of *A. santolina* and its isolated compounds in a first-time record.

## Materials and methods

The detailed procedures are described in the Supplementary online resource.

### Plant material

*Achillea santolina* L. flowering aerial parts were collected from a wild population growing in the vicinity of the Northern coast of Egypt, in April 2022. The plant’s identity was verified by Prof. Dr. Abd Haleem Abd El-Mogali, chief researcher, Flora and Phytotaxonomy Researches Department, Agriculture Museum, Giza, Egypt. The plant name was checked with The World Flora Online (https://www.worldfloraonline.org/taxon/wfo-0000058677, accessed 8 August 2023). Collection of the plant material complied with local, national, and international guidelines. At Faculty of Pharmacy, Cairo University’s Pharmacognosy Department Herbarium, a voucher specimen was deposited (registration number 10.04.2022I).

### Preparation and fractionation of *A. santolina* methanolic extract (AS) and fractions (MF and BF)

*A. santolina* flowering aerial parts (2 kg) were air-dried, powdered, then extracted with methanol (5 L x 4) till exhaustion at room temperature. The combined extracts were subjected to evaporation under reduced pressure at a temperature not exceeding 50 °C, yielding 98 g of dry residue. Two solvents (1 L x 8, each) of various polarities were used to partition the residue (55 g) in a suspension (in 300 mL of distilled water): methylene chloride and *n*-butanol saturated with water. The solvents were concentrated, yielding methylene chloride (MF) and butanol (BF) fractions weighing 18 and 22 g, respectively.

### HPLC analysis *of A. santolina* methanolic extract (AS)

HPLC analysis was conducted on Agilent 1260 infinity HPLC Series (Agilent, USA) with a quaternary pump, aKinetex^®^ 5 μm EVO C18 100 mm x 4.6 mm column (Phenomenex, USA), operated at 30 °C, and a ternary linear elution gradient with (A) HPLC grade water 0.2% H_3_PO_4_ (v/v), (B) methanol, and (C) acetonitrile at a flow rate of 0.2 mL/min. At λ 280 nm, a variable wavelength detector (VWD) was used (Agilent Application Note, Publication number 5991-3801EN, 2014). Three biological replicates were examined. Qualitative determination was accomplished by comparing peak retention times to those of the standard phenolics. While peak area measurement allowed for quantitative determination.

### Isolation of the major phenolics from the methylene chloride fraction (MF)

The methylene chloride fraction (10 g) was chromatographed on silica gel H 60 (250 g) using a vacuum liquid chromatography column (VLC) (10 L ҳ 12.5 D cm). Fractionation was achieved by adopting gradient elution, beginning with methylene chloride and increasing the polarity with ethyl acetate by 20% until 100% ethyl acetate, and then methanol by 10% increments until 100% pure methanol. The purification and isolation of each compound are recorded in the supplementary.

### Isolation of the major phenolics from the butanol fraction (BF)

Fractionation of the butanol fraction (BF) (15 g) was performed using polyamide column (25 L x 5 D cm) adopting gradient elution with 0–100% MeOH in water. The detailed procedures for isolation are recorded in the supplementary.

### Animals

Young male Wistar rats, weighing 40–60 g, were obtained from the Animal breeding Colony at National Organization for Drug Control and Research (NODCR, Egypt). The animals were kept in well-ventilated group housing with open polycarbonate cage with wire lid that held chow and water bottle, at pathology department, Faculty of Veterinary Medicine, Cairo University, Egypt. Rats were kept with unrestricted access to a well-balanced diet (vitamins mixture, 1%; minerals mixture, 4%; corn oil, 10%; sucrose, 20%; cellulose, 0.2%; casein, 10.5%; and starch, 54.3%) and water ad libitum throughout the experimental period. Controlled room temperature at 25 ± 1 °C; relative humidity at 54–68% and 12 h light/dark cycle were employed. The implemented experimental methodology was strictly adhered to the requirements established in the Guide for the Care and Use of Laboratory Animals and approved by Cairo University’s Institutional Animal Care and Use Committee (IACUC) (Vet CU 01122022581).

### Experimental protocol

Sixty-three rats were randomly allocated into nine experimental groups (*n* = 7): Control Group; served as negative control that received distilled water (DW) at the volume of 1 ml/rat orally via an intragastric tube once a day for 14 days. MSG Group, served as control positive where rats received DW at the volume of 0.5 mL/rats orally + 2 g/kg weight MSG dissolved in normal saline at the volume of 0.5 mL/rats orally via an intragastric tube once a day for 14 days^21^. Treated groups received MSG same way as MSG group along with various treatments as following: AS 200, 400 Groups; received *Achillea santolina* methanolic extract at doses of 200, 400 mg/kg, respectively. MF 200,400; received methylene chloride fraction of *Achillea santolina* extract with the same two doses and volume suspended in DW, BF 200, 400 received butanol fraction of *Achillea santolina* extract with the same two doses and volume suspended in DW, and standard group (Std): received Dextromethorphan 30 mg/kg at the volume of 0.5 mL/rats orally^6^.

### Samples collection and Preparation

One day post the last dose, rats were anesthetized using ketamine 90 mg i.p and Blood was drawn from the Orbital sinus^22^ followed by euthanizing of all rats^22^. Collected blood was centrifuged and obtained serum was used for lactate dehydrogenase (LDH) assays. The whole brains were expunged, washed with cold saline, wiped with filter paper, and split into two portions(hemispheres): one hemisphere was used for histopathological examinations following formalin fixation, however the other hemisphere was snap-frozen in liquid nitrogen and kept at − 80 °C to be used for biochemical assessment (MDA, GSH and TNF-α) and for RNA extraction. Samples were assayed in triplicates^2^.

### Oxidative and inflammatory biomarkers evaluation

The malondialdehyde (MDA) levels and reduced glutathione (GSH) activity were measured to evaluate oxidative stress levels in the brain. Brain homogenate was prepared using a 50 mM potassium phosphate buffer with a pH of 7.5 for MDA measurement. While for GSH measurement, 1 mM EDTA was added. The homogenate was then centrifuged at 4000 rpm for 15 min to obtain the supernatant, which was used to measure MDA and GSH levels using colorimetric kits (MD 25 29 and GR 25 11, Biodiagnostic Co. Dokki, Giza, Egypt) following^23^.

The inflammatory biomarker TNF-α was quantified using a rat-specific ELISA kit (ELK1387, ELK Biotechnology, China) according to the manufacturer’s guidelines. The activity of lactate dehydrogenase (LDH) in the serum was estimated using commercial kits (264 002, 260 002, E.C.1.1.1.27, Spectrum Diagnostics Co., Cairo, Egypt) following the previously outlined protocols^24,25^.

### Histopathological examination

Brain specimens were preserved in 10% neutral buffered formalin followed by processing in increasing concentrations of ethanol and xylene. The tissues were embedded in paraffin wax and sectioned into 3–4 μm thick sections using a rotary microtome. They were then stained with haematoxylin and eosin.

### Immunohistochemical examination

Tumor necrosis factor alpha was detected in paraffin embedded tissue by immunoperoxidase technique. Briefly, tissue sections were deparaffinized and then incubated in citrate buffer PH 6 for antigen retrieval. Anti- TNF-alpha antibodies (Santa Cruz, USA) were applied to slides overnight followed by washing and application of secondary antibodies and DAB according to manufacturer protocol of universal kit (Bio SB, USA). Area percent of positive staining was measured by Image J software in 3 sections/ group at 200X magnification.

### Quantitative real time-PCR (qRT-PCR)

Following the manufacturer’s recommendations samples were homogenized with liquid nitrogen, and the total RNA was extracted using the Qiagen RNeasy Mini Kit. To remove any DNA contamination, DNase I (Fermentas) was used. A NanoDrop spectrophotometer was used to measure the concentration and purity of the isolated RNA. Reverse transcription polymerase chain reaction (RT-PCR) was done using the RevertAid First Strand cDNA Synthesis Kit, adhering to the manufacturer’s instructions. Quantitative real-time PCR (RT-qPCR) was carried out with the Luminaries Color HiGreen Low ROX qPCR Master kit (Thermo Scientific, K0371), following the recommended guidelines.

Beta actin mRNA levels were used to normalize all values. The cDNA underwent amplification through 35 cycles, consisting of denaturation at 95 °C for 40 s, annealing at 58 °C for 40 s, and extension at 72 °C for 40 s. The experiments were conducted in duplicate plates, and the cycle threshold (Ct) values were utilized to compute the gene/GAPDH ratio using a calibrator value set to 1.0. The normalized expression ratio was calculated with the ΔΔCt method. The primer sets for detecting the mRNA levels of COX-2, IL-1β and IL-10 are shown in Supplementary Table S1.

### Statistical analysis

All *in vitro* determinations were performed in triplicate and reported as mean ± standard deviation. The IC_50_ values were determined by transforming the concentrations to logarithmic form and applying a non-linear regression equation for inhibitors (log inhibitor) versus the normalized response-variable slope equation. *In vitro* studies were analyzed using one-way ANOVA and Tukey’s test, with p values under < 0.05 regarded as significant. Multivariate ANOVA was employed for quantitative *in vivo* studies to find the variabilty between groups with LDS post hoc test and expressed as mean ± standard deviation, *n* = 7. While, for qualitative *in vivo* studies as PCR expression and area percent of immunohistochemistry analysis a nonparametric analysis using Kruskal-Wallis test was employed and results expressed as mean ± standard error, *n* = 7. (*) Indicates significant difference compared to MSG group at *P* ≤ 0.05 and (**) Indicates high significant difference compared to MSG group at *P* ≤ 0.005. (#) Indicates significant compared to control group at *P* ≤ 0.05 and (##) Indicates high significant compared to control group at *P* ≤ 0.005. All figures and statistical analyses were generated with GraphPad Prism version 8.0^®^ for Windows (GraphPad Software, San Diego, California USA).

### System preparation and molecular docking

The crystal structures of Human Heme Oxygenase-1 (HO-1), Human 5-lipoxygenase (5-LOX), Kelch-like ECH-associated protein (Keap1), and Cyclooxygenase-2 (COX-2) were retrieved from the protein data bank with code 3HOK, 3V99, 4L7B,, 6COX and prepared using UCSF Chimera. The detailed explanation is recorded in the supplementary.

### Molecular docking

The structures of the extracted compounds were drawn using ChemOffice tool (ChemDraw 16.0) assigned with proper 2D orientation. The detailed method is described in the supplementary.

### Molecular dynamic (MD) simulations

The AMBER 18 package included the PMEMD GPU engine, was utilized to run the MD simulations for every system. The procedure is recorded in the supplementary.

## Results and discussion

### Phytochemical assessment of *A. santolina* methanolic extract (AS), methylene chloride (MF) and butanol (BF) fractions

#### Total phenolic (TPC) and total flavonoid (TFC) contents determination

**Fig. 1:**
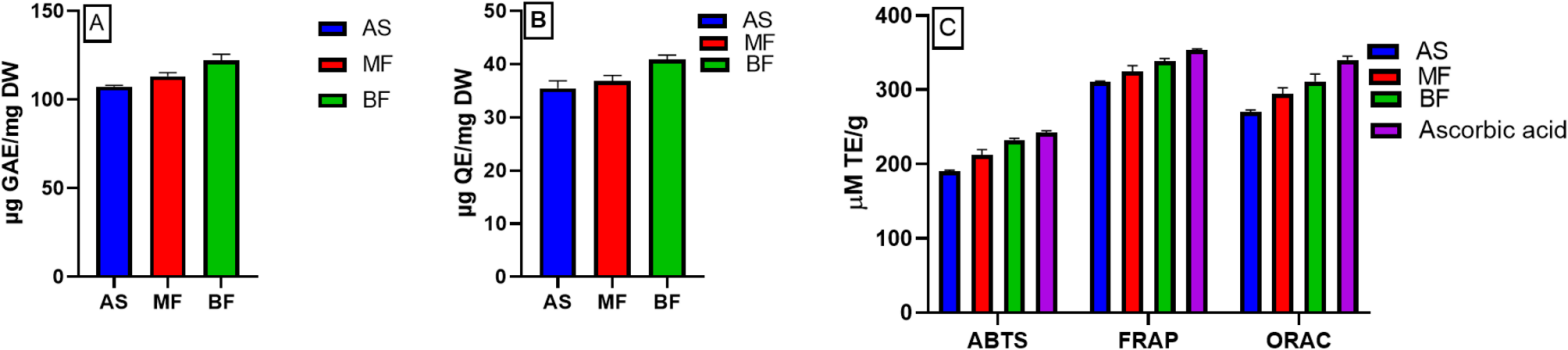
(A) Total phenolic content (TPC) (B) total flavonoid content (TFC) (C) antioxidant activity (ABTS, FRAP and ORAC assays) of *A. santolina* methanolic extract (AS), methylene chloride (MF), and butanol (BF) fractions. µM: micromolar; AS: *A. santolina* methanolic extract; BF: butanol fraction; GAE: gallic acid equivalent; TPC: total phenolic content; QE, quercetin equivalent; TE: Trolox equivalent; TFC, total flavonoid content; MF: methylene chloride fraction. Data are represented as mean ± standard error of three replicates.

The total phenolic (TPC) and flavonoid (TFC) contents of AS, MF and BF were evaluated (Fig. 1A and B). Herein, the TPC of AS was calculated as 107.40 ± 0.46 µg gallic acid equivalent (GAE)/mg dried weight (DW). The TPC in *A. santolina* was previously determined using a different standard for phenolic content calculation^16^. The result was nearly similar to a previous study conducted on 70% ethanolic extract (TPC 104.66 ± 4.39 s µg GAE/mg DW)^15^. The BF exhibited the highest TPC of all tested samples (122.2 ± 2.00 µg GAE/mg DW). The TFC of AS was 35.46 ± 0.83 µg quercetin equivalent (QE)/mg DW, which was higher than that previously determined (9.7 ± 0.33 µg QE/mg DW)^16^. *A. santolina* flavonoid content was also reported by Ardestani & Yazdanparast^15^, but using a different standard to calculate it. The highest TFC of all tested samples was detected in BF 40.85 ± 0.51 µg QE/mg DW.

#### *In vitro* antioxidant activity

Oxidative stress is an important factor in the development of inflammatory and neurodegenerative disorders^20^. Thus, natural antioxidants (phenolic compounds) have been suggested as therapeutic strategy for prevention and treatment neurological diseases^26^. The antioxidant potential of AS, MF and BF was determined using various *in vitro* assays *viz.;* radical scavenging activity (ABTS), redox potential (FRAP) and ORAC (Fig. 1C). The highest antioxidant potential was recorded by BF with values of 232.50 ± 1.40, 338.90 ± 1.89 and 311.30 ± 5.60 micromolar (μM) Trolox equivalent (TE)/g representing 95.91, 95.89 and 91.50% of ascorbic acid antioxidant activity in ABTS, FRAP and ORAC, respectively. The high antioxidant potential of BF could be correlated to its high TPC and TFC^27^. The results were in accordance with the previously determined antioxidant potential of *A. santolina*^15,16^. To our knowledge, this is the first study on the ABTS activity of *A. santolina* methanolic extract and its fractions.

#### HPLC analysis of*A. santolina* methanolic extract (AS)

HPLC analysis identified and quantified 22 phenolic compounds (6 phenolic acids and 16 flavonoids) using the available standards (Table 1, Fig. S1). The results showed that *A. santolina* methanolic extract was rich in phenolics matching previous studies^28^. The phenolic acids were mainly benzoic acid (gallic, protocatechuic and vanillic acids) and cinnamic acid derivatives (chlorogenic, caffeic and ferulic acids). The flavonoids were represented by flavone aglycones (luteolin and apigenin), flavone-C-glycosides (orientin, isoorientin, vitexin and isovitexin), flavonol aglycones (myricetin, quercetin, kaempferol), flavonol-*O*-glycoside (rutin, isoquercitrin, quercitrin, kaempferol-3-*O*-glucoside), *O*-methylated flavonols (isorhamnetin and rhamnetin) and methylated flavonol-*O*-glycoside (isorhamnetin-3-*O*-glucoside). Luteolin, kaempferol 3-*O*-glucoside (astragalin), isovitexin and kaempferol were the major phenolics detected corresponding to 506.94 ± 1.09, 318.45 ± 056, 240.81 ± 1.97, and 140.47 ± 0.68 mg /100 g respectively. The results matched previous findings detecting luteolin as the most abundant flavonoid in *Achillea* species^16^. In addition, the flavonoid profile was in accordance with previous study conducted on *A. santolina* identifying luteolin, apigenin, isorhamnetin, quercetin, and kaempferol in its ethyl acetate fraction, where luteolin was the predominant constituent detected^29^. There are numerous health-promoting properties of flavonoids and phenolic acids. They have a variety of neuroprotective effects on the brain, such as protecting neurons against neurotoxins and reducing neuronal inflammation, which enhances cognition, memory, and learning^30^. This neuroprotective potential is mainly due to their capacity to control inflammatory reactions through reduction of the pro-inflammatory cytokines expression (IL-6, IL-1β, TNF-α and COX-2), down regulating inflammatory markers and prevention of neural impairment^31^. Luteolin showed anti-inflammatory effects on microglia; in fact, in LPS-induced BV2 microglia cells, luteolin drastically decreased iNOS and COX-2 expression, downregulated pro-inflammatory cytokines, and increased NO and prostaglandin E2 production^32^. Apigenin was also shown to have an inhibitory effect on inflammatory markers and neuroprotective potential. Apigenin and luteolin (10–50 µM) dramatically reduced the expression of CD40 produced by IFN-γ and, concurrently, inhibited the pro-inflammatory cytokines TNF-α and IL-6 release in both cultured and murine-derived microglia cell lines. Additionally, the inactivation of STAT1 was a mediator of the effects brought on by these flavones^33^. According to their anti-inflammatory action, other flavonols, such as rhamnetin^34^, kaempferol^35^, and quercetin^36^, also exhibit neuroprotective properties. Luteolin down-regulated TLR-4, NF-κB, p38- MAPK, JNK, and AKT in BV2 microglial cells after LPS produced inflammatory mediators^35^.

**Table 1:** Quantification of phenolic compounds identified in *A. santolina* methanolic extract (AS) using HPLC.

Identified PA	RT (min)	*Conc. (mg /100 g ± SD)
Gallic acid	1.443	96.80 ± 0.68
Protocatechuic acid	1.767	55.10 ± 0.34
Chlorogenic acid	2.550	31.12 ± 0.44
Vanillic acid	3.387	45.79 ± 0.68
Caffeic acid	4.154	3.36 ± 0.55
Ferulic acid	6.647	32.37 ± 1.97
Orientin	7.144	20.36 ± 0.58
Isoorientin	8.464	10.98 ± 0.36
vitexin	8.872	14.89 ± 0.99
Isovitexin	9.247	240.81 ± 1.97
Rutin	13.501	24.50 ± 0.12
Isoquercitrin	14.766	15.12 ± 0.78
Quercitrin	15.858	31.56 ± 0.23
Kaempferol-3-*O*-glucoside	16.920	318.45 ± 056
Isorhamnetin-3-*O*-glucoside	17.273	5.31 ± 0.11
Myricetin	18.311	2.29 ± 0.05
Luteolin	18.739	506.94 ± 1.09
Quercetin	20.548	39.78 ± 1.97
Apigenin	21.225	29.36 ± 0.58
Kaempferol	22.510	140.47 ± 0.68
isorhamnetin	22.913	11.37 ± 0.23
Rhamnetin	26.053	33.60 ± 0.82

RT; retention time in minutes, SD: standard deviation.

*Average concentration of three HPLC determinations.

#### Identification of the isolated compounds (C1-C4)

**Fig. 2:**
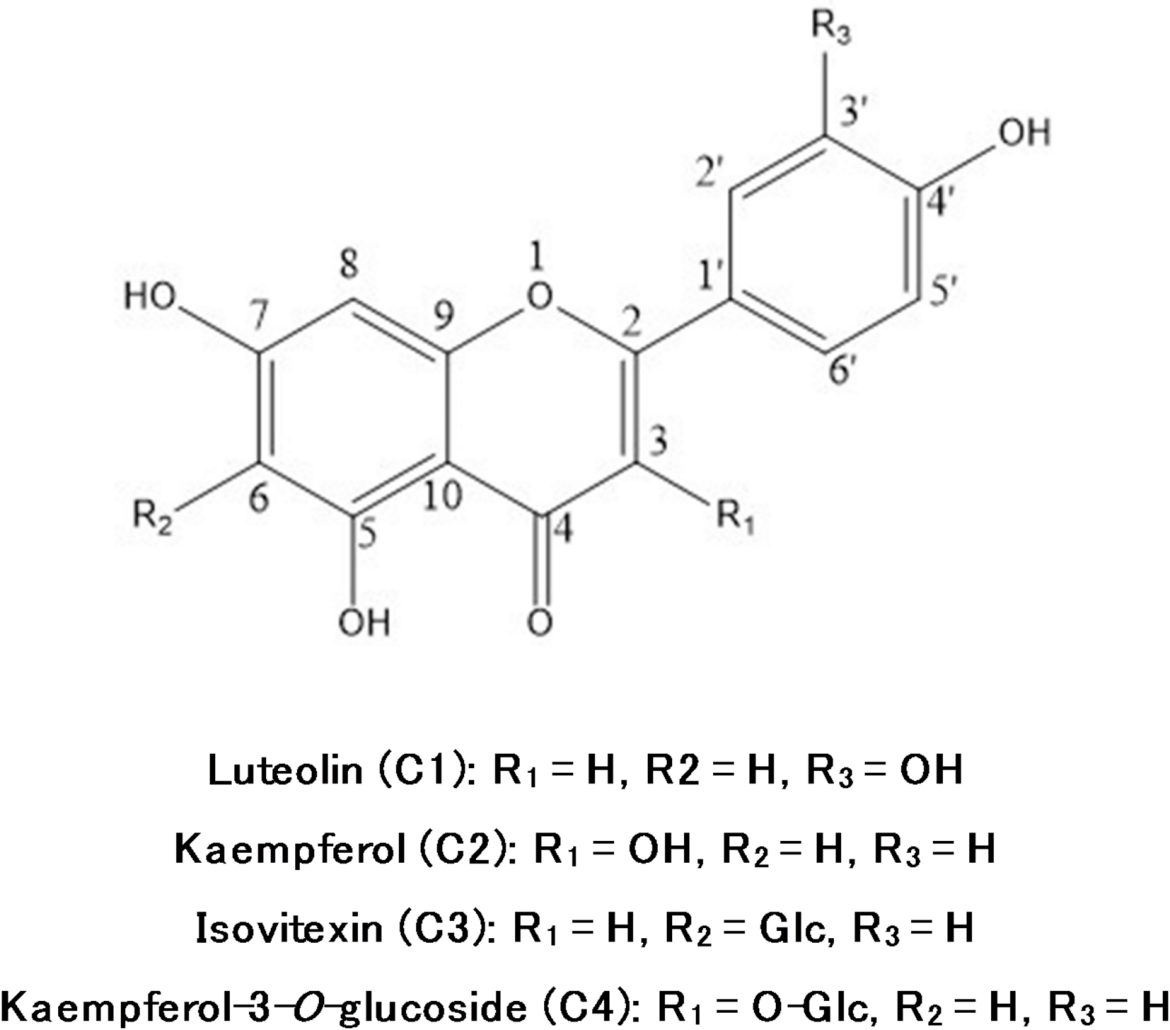
Structure of the isolated compounds.

Four isolated compounds were identified through their Rf values, chemical methods (acid hydrolysis and ferric chloride oxidative hydrolysis) and spectral data (UV,^1^HNMR and^13^C-NMR) (see supplementary material) and direct comparison to the literature^37–40^ as luteolin (C1), kaempferol (C2), isovitexin (C3) and kaempferol-3-*O*-glucoside (astragalin) (C4). Structures of the identified compounds are represented in Fig. 2.

### *In vitro *anti-inflammatory activity of the isolated compounds (C1-C4)

**Fig. 3:**
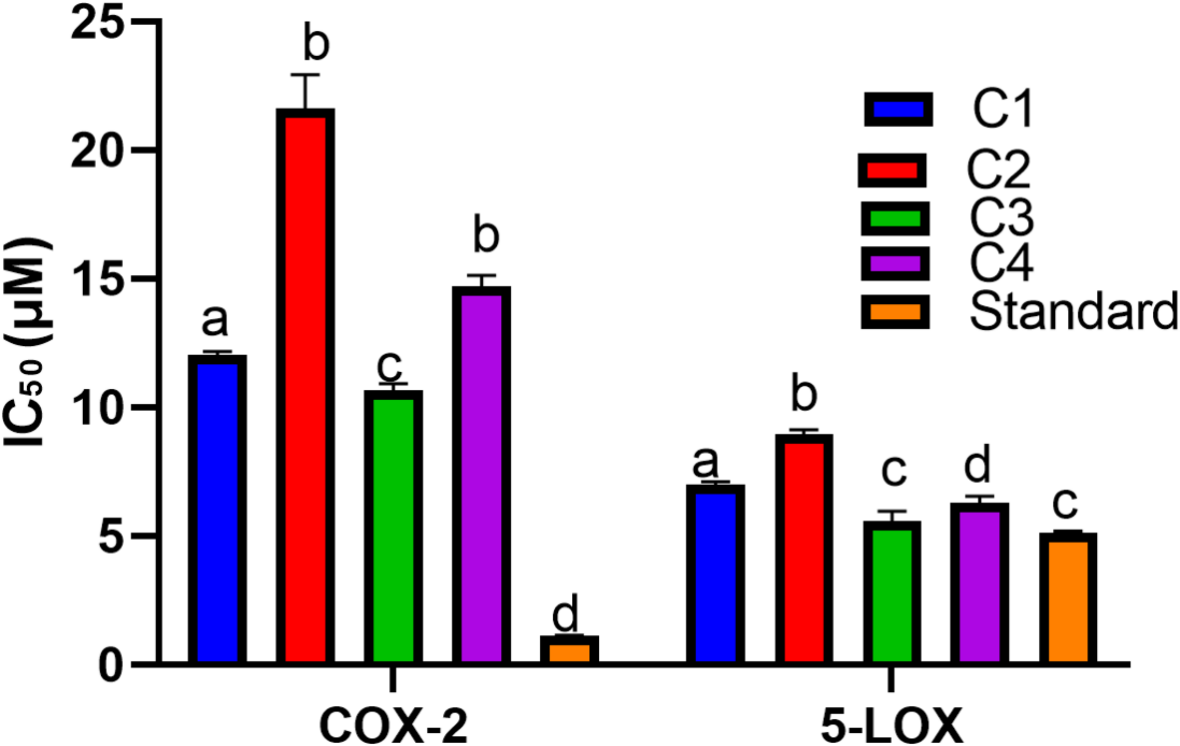
*In vitro* (A) COX-2 and (B) 5-LOX inhibitory potentials of the isolated compounds (C1-C4).

The inhibitory potential of the isolated compounds (C1-C4) on the inflammatory enzymes COX-2 and 5-LOX was examined (Fig. 3) in order to understand their capabilities to develop effective intervention for the prevention of inflammatory-related diseases. The isolated compounds displayed noteworthy COX-2 inhibitory activities in the range of 10.67 ± 0.14 to 21.61 ± 0.76 µM relative to Celecoxib [reference standard (IC_50_ 1.13 ± 0.01 µM)].

Herein, isovitexin (C3) exhibited the most powerful COX-2 inhibitory potentials followed by luteolin [C1 (IC_50_ 12.04 ± 0.08)]. The results were in accordance with previous findings studying the COX-2 inhibitory potentials of the isolated compounds^41^. In addition, the isolated compounds (C1-C4) showed high 5-LOX inhibitory potentials varying from 5.57 ± 0.23 to 8.96 ± 0.10 µM relative to the Zileuton [reference standard (IC_50_ 5.13 ± 0.04 µM)].

Isovitexin (C3) showed the highest inhibitory potential against 5-LOX followed by astragalin [C4 (IC_50_ 6.28 ± 0.16)]. In previous studies, the isolated compounds displayed high 5-LOX inhibitory potentials^41^.

This study investigated the isolated compounds’ anti-inflammatory potential and concluded that they may be capable of combating inflammation, which contributes to the exacerbation of neurodegenerative diseases. These findings are consistent with earlier research showing the anti-inflammatory properties of flavonoids^42^.

µM: micromolar; Data are expressed as mean ± standard error of three replicates; Different letters on the bar indicate significant differences at *P* < 0.0001 with Tukey’s test. Standards: Celecoxib (COX 2) and Zileuton (5-LOX) are serving as positive controls.

### Oxidative and inflammatory biomarkers evaluation

Although the toxicity of various *Achillea* species has been studied, specific LD₅₀ values for Achillea santolina and its individual fractions remain limited. The LD₅₀ for the whole plant extract has been documented as 889 mg/kg^43^. Further research is needed to evaluate the toxicity of its distinct fractions and to determine whether their toxicological profiles differ. Several studies have highlighted the biological activities of *A. santolina* extract, demonstrating significant effects at doses of 200 mg/kg and 400 mg/kg, particularly in anti-inflammatory and immunomodulatory responses^18^. Additionally, the extract has shown antioxidant properties and hypoglycemic activity at a dose of 250 mg/kg^44^.

The well-established methods for using Monosodium glutamate, as neurotoxicity model administrated it intraperitoneally or SC at dosed for 2–5 g/kg for a week^21^ or orally at same doses for longer period up to one month^7^. In this study MSG was given orally at the lower dose of 2 g/kg for 14 days for animal welfare. The administration of MSG induced adverse effects on both oxidative stress and inflammatory biomarkers. It led to a significant increase in levels of MDA (a marker of lipid peroxidation), TNF alpha (a pro-inflammatory cytokine), and LDH (an indicator of cellular damage). Additionally, the activity of GSH (glutathione, an antioxidant) was reduced compared to the control group. Similarly,^7^ recorded MSG associated neurotoxicity which was corelated to decreasing cyclic AMP-activated protein kinase (AMPK) activity and increasing cholinesterase (ChE) levels in neurons.

**Fig. 4:**
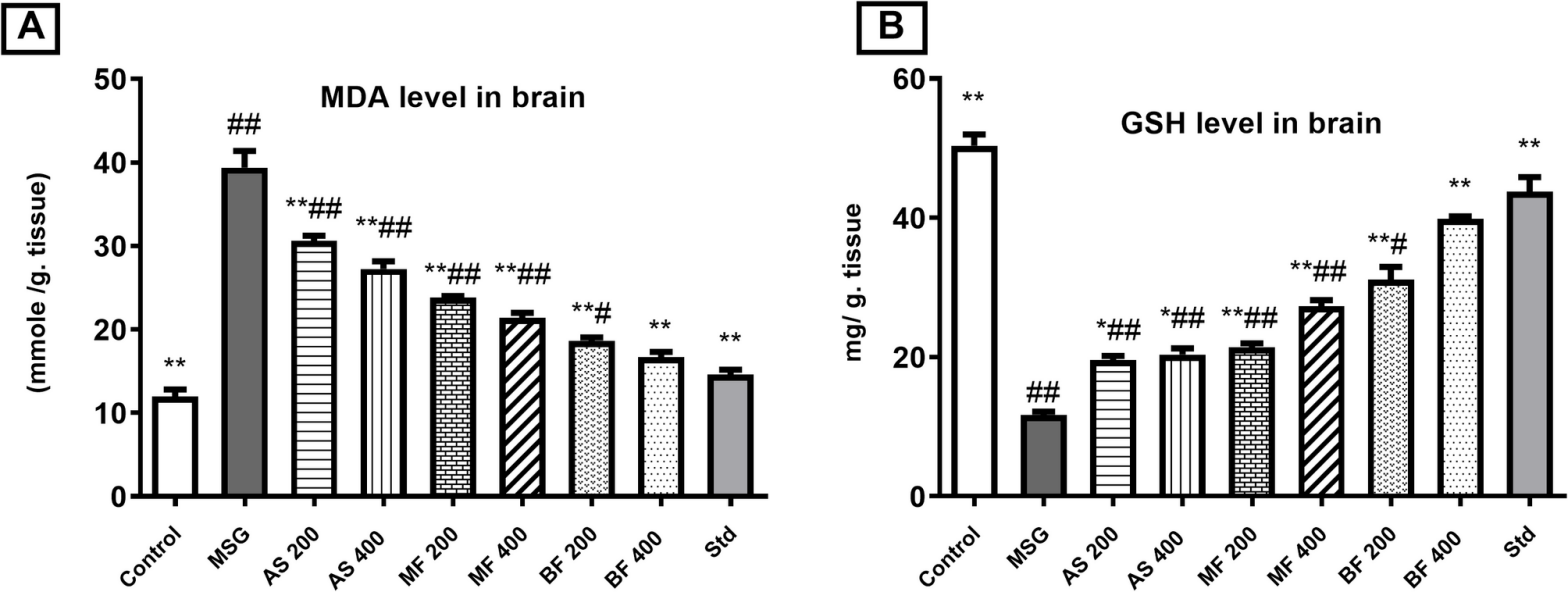
Oxidative biomarkers: (A) brain MDA level; (B) brain GSH activity. Results are displayed as mean ± SD, (*n* = 7), (#) and (##) indicate significant distinctions in comparison to the control group at *P* < 0.05 and < 0.005, respectively., whereas (*) and (**) indicate significant distinctions in comparison to the MSG group at *P* < 0.05 and < 0.005, respectively.

**Fig. 5:**
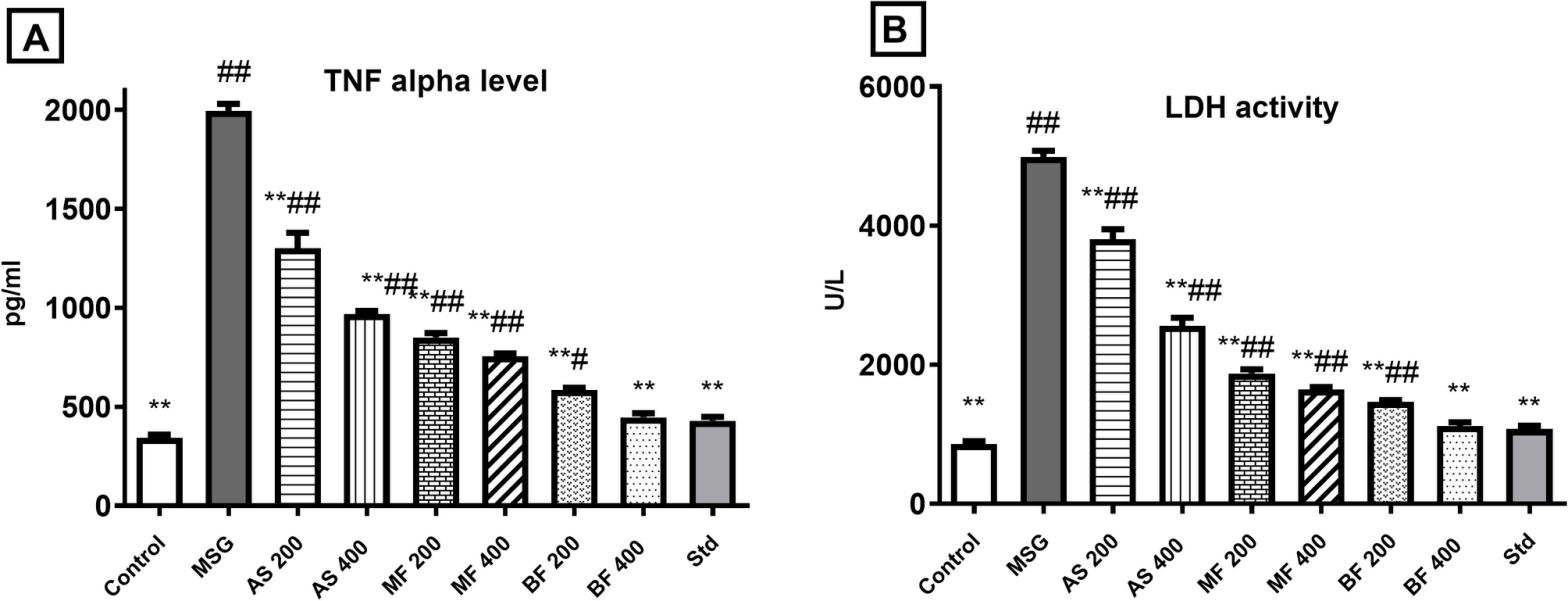
Inflammatory biomarkers: (A) TNF alpha level: (B) LDH activity. Results are displayed as mean ± SD, (*n* = 7), (#) and (##) indicate significant distinctions in comparison to the control group at *P* < 0.05 and < 0.005, respectively., whereas (*) and (**) indicate significant distinctions in comparison to the MSG group at *P* < 0.05 and < 0.005, respectively.

However, all groups treated with *A. santolina* and its fractions (AS, MF and BF) showed improvements in these effects. They exhibited decreased MDA, TNF alpha, and LDH levels, indicating a reduction in oxidative stress and inflammation. Moreover, the activity of GSH was increased compared to the MSG group, suggesting enhanced antioxidant capacity^23,24^. Among the different treatment groups, the BF (200, 400) groups, particularly at the dose of 400 mg/kg, showed the most significant enhancement in antioxidant and anti-inflammatory biomarkers. The MF groups followed, and then the AS groups, as shown in Figs. 4 and 5. The difference in the enhancement of the antioxidant and anti-inflammatory biomarkers by the different fractions tested followed the same pattern with their respective TPC, TFC and *in vitro* antioxidant potential. As a result, BF was the most significant owing to its high content of phenolics and flavonoids as well as its high antioxidant power^45^.

The former results support the *in vitro* COX-2 and 5-LOX inhibitory activities shown earlier in this study **(**Fig. 3**).** Similarly, *A. santolina* extract was reported to exhibit a high antioxidant, free radical scavenging and explained that by inhibiting Fe^2+^/ascorbate induced lipid peroxidation^15^. Moreover, it could be attributed to phenolic and flavonoid contents of *A. santolina* as suggested by previously,^14,46^ indicating that *Achillea* antioxidant and anti-inflammatory activities correlated mainly to its content of the essential oil, proazulenes other sesquiterpene lactones, dicaffeoylquinic acids, camphene, limonene and apigenin. Herein, the compounds detected and isolated from *A. santolina* extract and fractions have been extensively researched for their potential to counteract oxidative stress and inflammation. Studies have shown that these phenolic compounds, which are commonly found in plant-based foods and herbal extracts, possess robust antioxidant and anti-inflammatory properties, making them potentially beneficial for overall health^47^. For instance, gallic acid has been found to neutralize free radicals and decrease the production of pro-inflammatory cytokines, such as TNF-α and IL-1β, by blocking the activation of key signaling pathways, such as NF-κB and MAPK^48^. Furthermore, protocatechuic acid has been reported to inhibit the activity of enzymes responsible for the production ROS, thereby lowering oxidative stress and providing a healthier cellular environment^49^. Flavonoids, such as quercetin and kaempferol, luteolin and apigenin have also been reported to modify several molecular pathways, including NF-κB and MAPK, which play a fundamental role in regulating the inflammatory response^50^. Quercetin has been found to inhibit the activation of NF-κB by blocking the degradation of IκB and decreasing the translocation of NF-κB to the nucleus, ultimately leading to a decline in the pro-inflammatory cytokines production^51^. On the other hand, Kaempferol inhibited the activation of MAPK signaling pathways, including ERK and JNK, which are responsible in the regulation of inflammatory responses^52^. Isovitexin have also been found to inhibit the activation of NF-κB and MAPK signaling pathways, resulting in a reduction in the formation of pro-inflammatory cytokines^53^. Orientin inhibited the production TNF-α and IL-6 and the activation of NF-κB^54^. Isoorientin reduced neuroinflammation by inhibition of the ROS-related MAPK/NF-κB signaling pathway^55^.

### Histopathological findings

**Fig. 6:**
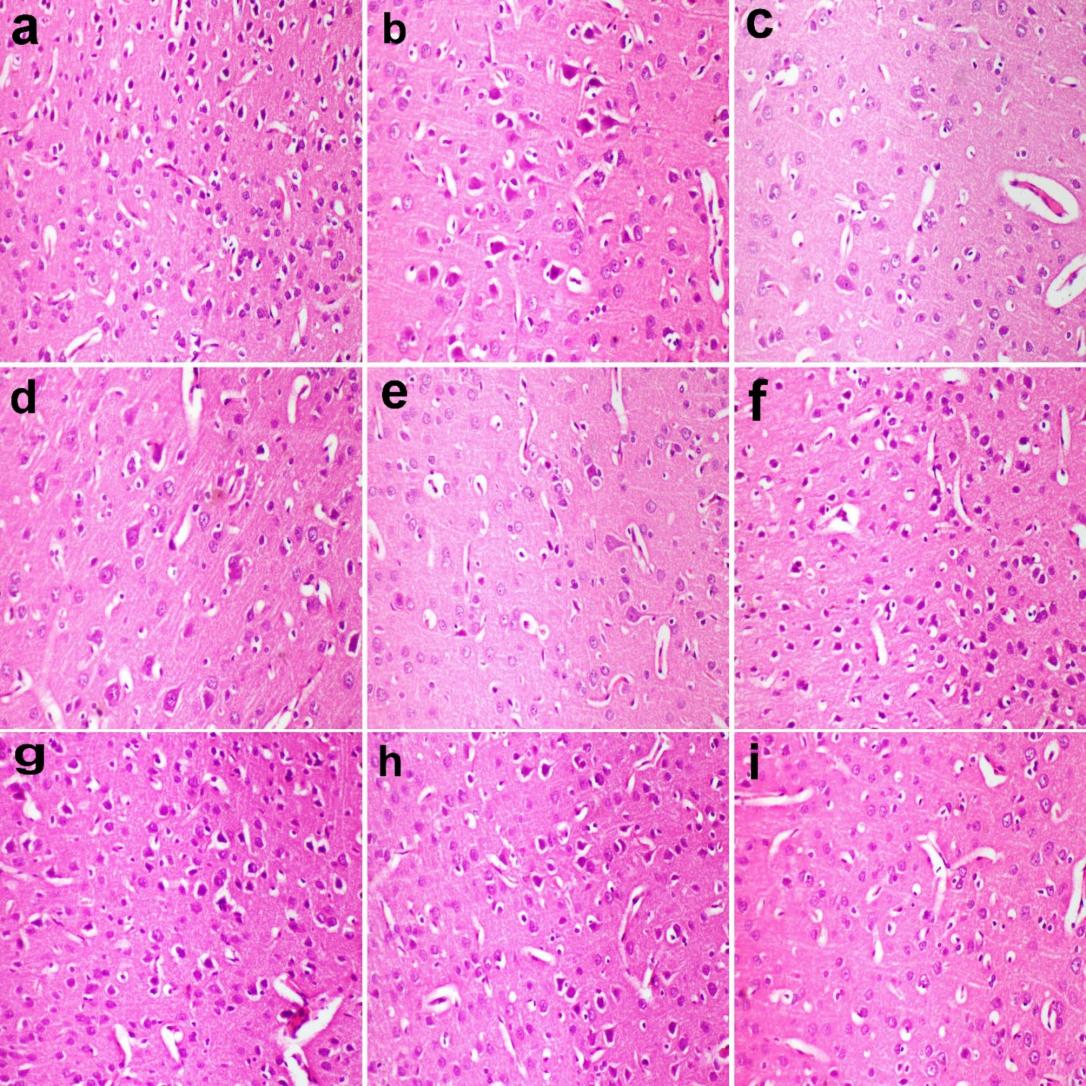
cerebral cortex, rats. (a) normal histological structure in control group. (b) degenerated neurons and gliosis in MSG group. (c) mild neuronal degeneration and gliosis in AS 200 and (d) AS 400 groups. (e) moderate neuronal degeneration and gliosis in polar 200, (f) polar AS 400, and (g) nonpolar AS 200 groups. (h) mild neuronal degeneration and gliosis in nonpolar AS 400 and (i) dextromethorphan groups. H and stain X 200.

Microscopy of the cerebral cortex revealed normal histological structure in control group (Fig. 6a). In MSG group, the pyramidal cells in the cerebral cortex were degenerated and showed central chromatolysis. It was associated with gliosis (Fig. 6b). In the treated groups, there was an improvement in the lesions observed compared to MSG group (Fig. 6c-i).

**Fig. 7:**
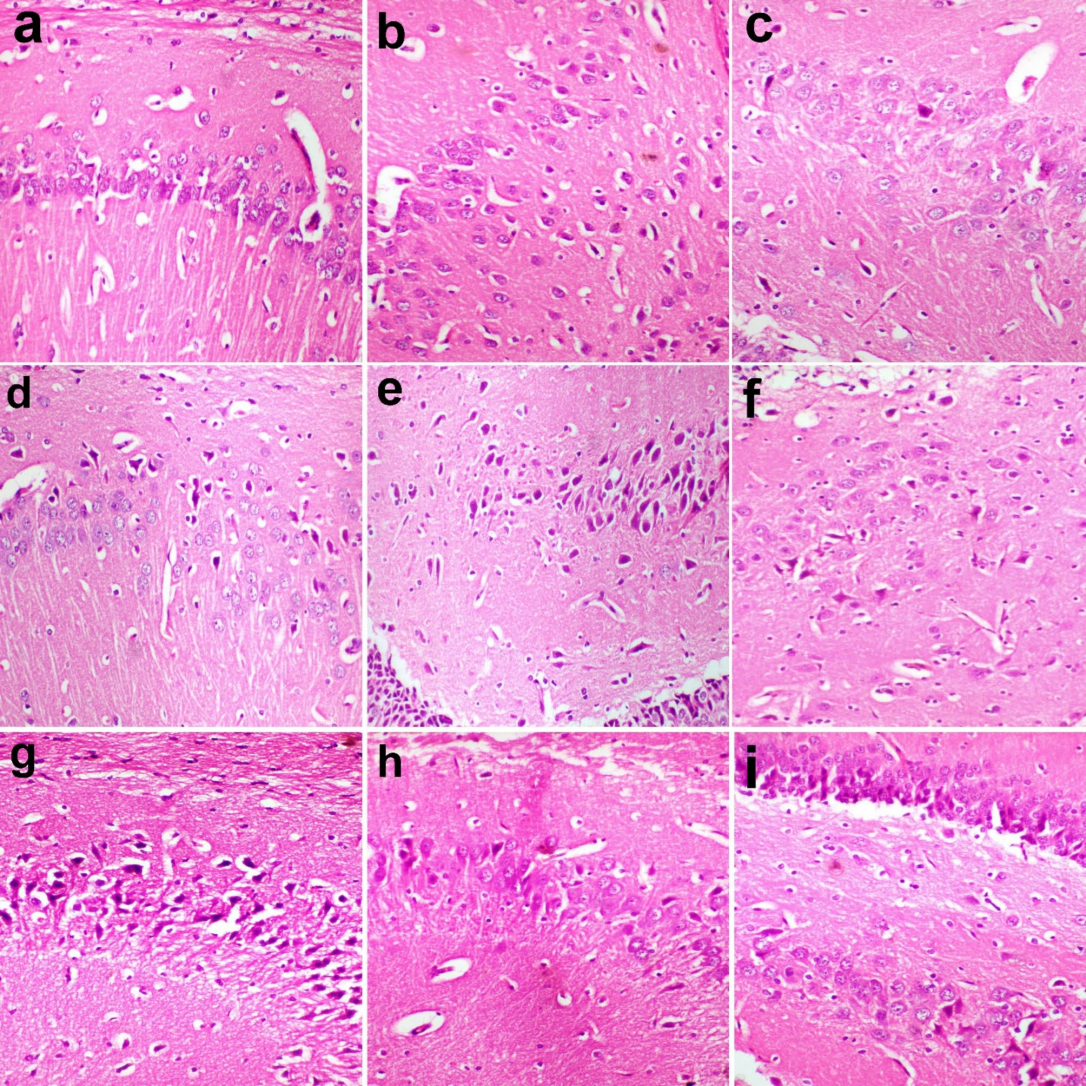
brain hippocampus, rat. (a) normal histological structure in control group, (b)neuronal degeneration and gliosis in MSG group, (c) neuronal degeneration and gliosis was mild in AS 200 and (d) moderate in AS 400 groups. (e) neuronal degeneration and gliosis was moderate in polar 200, (f) mild polar AS 400, (g) moderate in nonpolar AS 200 groups, and (h) mild in nonpolar AS 400 and (i) dextromethorphan groups. H and stain X 200.

Microscopy of hippocampus revealed normal histological structure in control group (Fig. 7a). In MSG group, hippocampus histopathology showed decreased cellular density in Cornu Ammonis accompanied by gliosis (Fig. 7b). Neuronal degeneration and gliosis were mild in AS 200, moderate in AS 400, moderate in MF and BF at dose of 200 mg/kg, and mild in MF and BF at dose of 400 mg/kg (Fig. 7c-i).

### Immunohistochemical findings

**Fig. 8:**
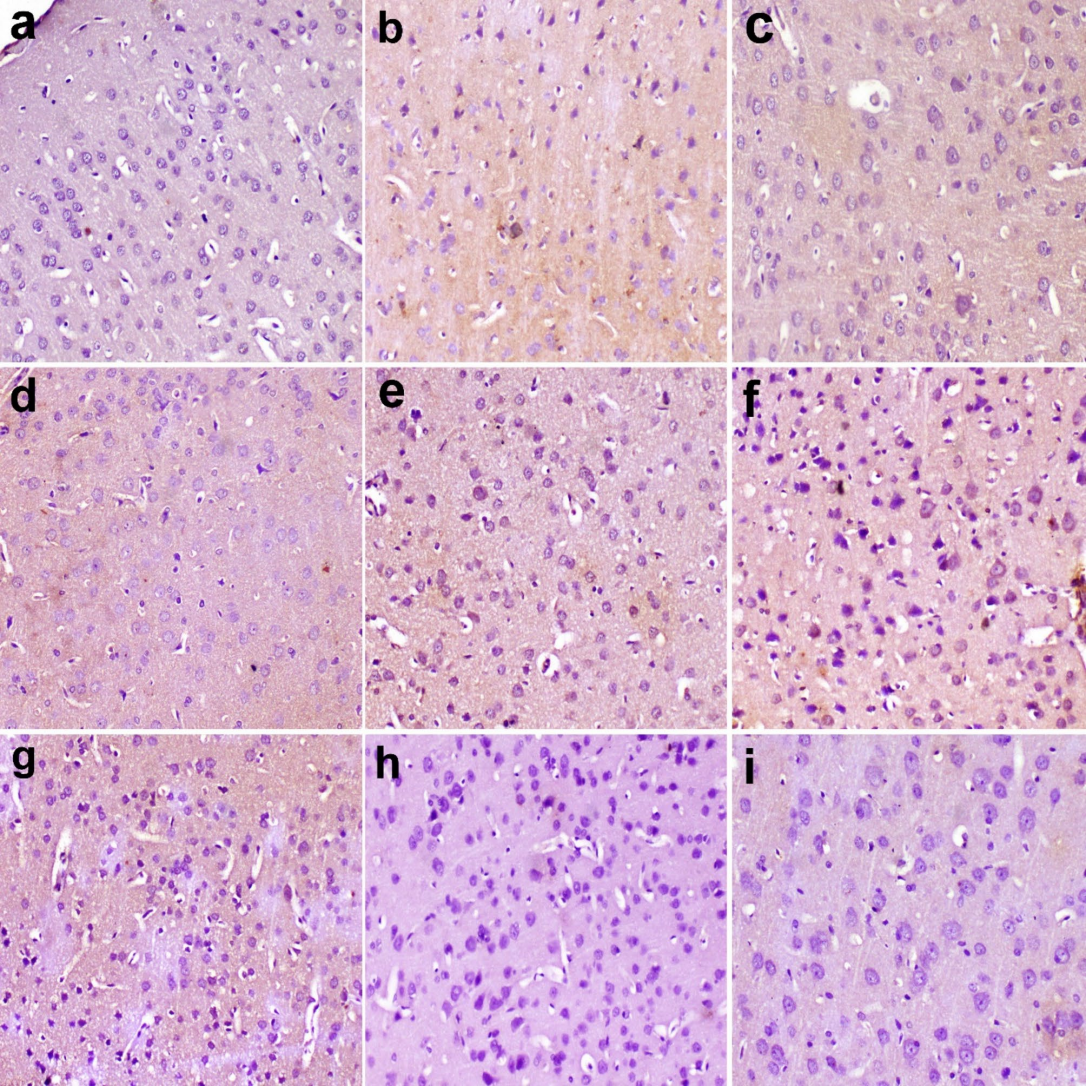
Immunohistochemistry of TNF-α in cerebral cortex. (a) no expression was observed in control group. (b) strong expression in the neurons of MSG group. (c) mild expression in AS 200 group. (d) moderate expression in As 400 group. (e) moderate expression in polar AS 200, (f) polar As 400 groups and (g) non polar As 200 groups. (h) weak expression in nonpolar As 400, and (I) dextromethorphan groups. Immunoperoxidase and hematoxylin counterstain X 200.

**Fig. 9:**
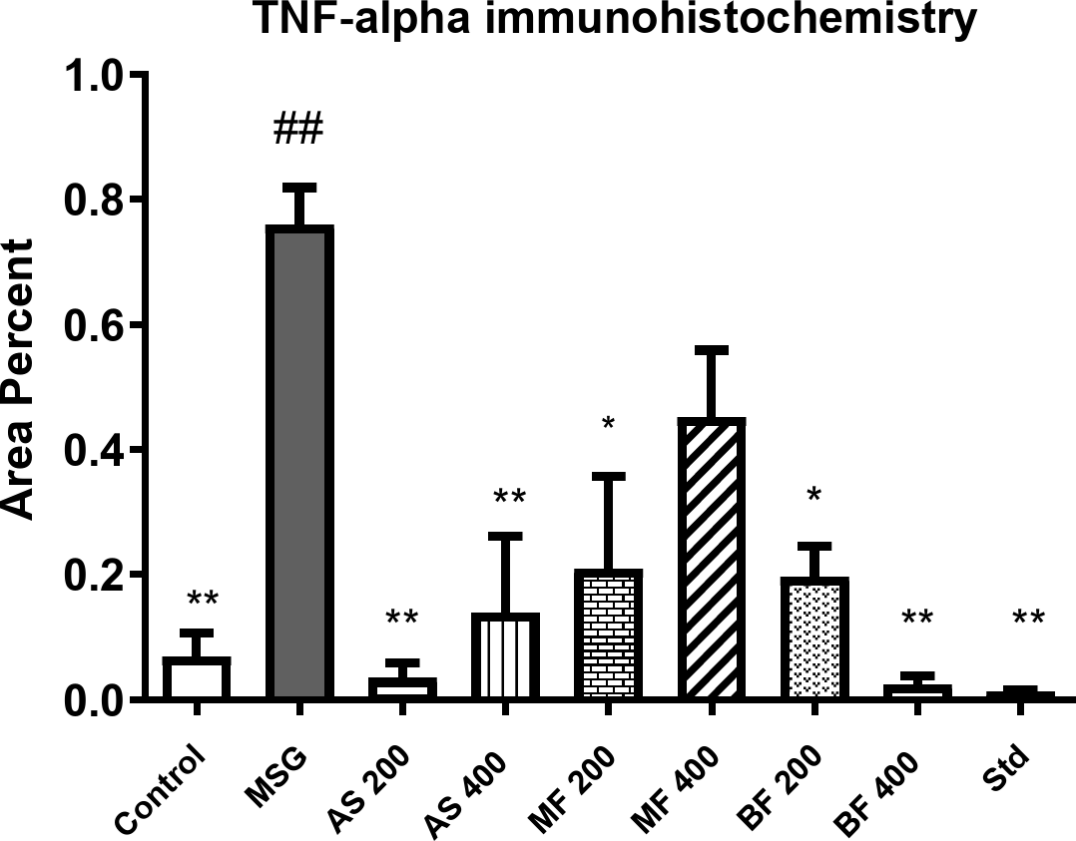
Area percent of TNF-alpha immunohistochemistry in different groups. Data are presented as mean values ± SE, (*n* = 7), (#) and (##) indicate significant distinctions in comparison to the control group at *P* < 0.05 and < 0.005, respectively, whereas (*) and (**) indicate significant distinctions in comparison to the MSG group at *P* < 0.05 and < 0.005, respectively.

TNF-α expression was minimal in the different regions of the brain in the control group (Fig. 8a). However, it was expressed in the pyramidal neurons of the cerebral cortex in MSG group and treated groups (Fig. 8b-i). The area percent of TNF-α expression was high in MSG group but decreased in treated groups. It recorded the lowest percent in BF 400 group. (Fig. 9).

### Quantitative real time-PCR (qRT-PCR)

**Fig. 10:**
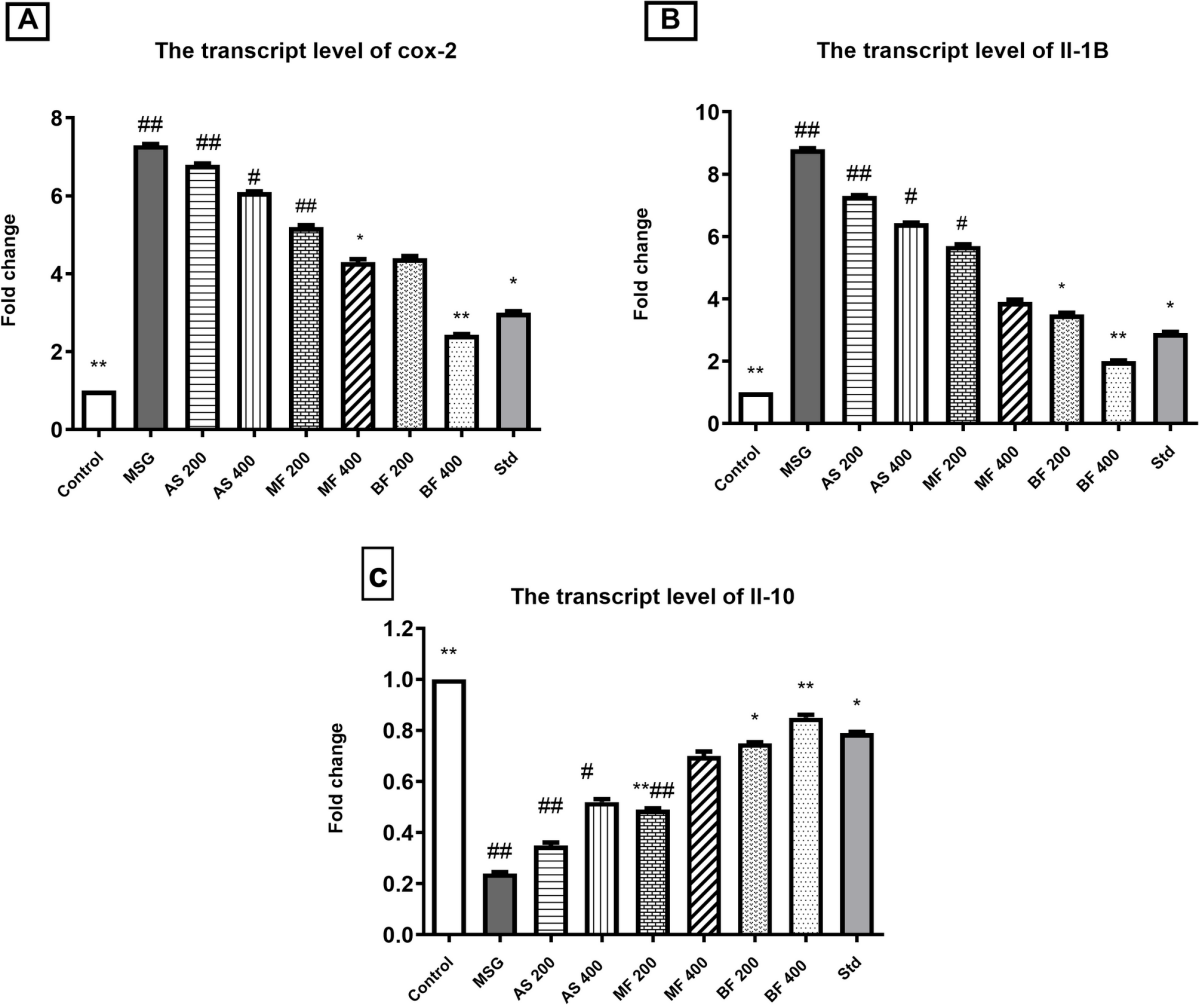
The mRNA expression rate of (A) cox-2; (B) IL-1B; C)IL-10. Data are presented as mean values ± SE, (*n* = 7), (#) and (##) indicate significant distinctions in comparison to the control group at *P* < 0.05 and < 0.005, respectively, whereas (*) and (**) indicate significant distinctions in comparison to the MSG group at *P* < 0.05 and < 0.005, respectively.

The control positive group recorded significant upregulations of cox-2, IL1B and IL-10. However, all treatments significantly ameliorated the hazard effect of MSG interestingly the polar (MF) and non-polar (BF) fractions of the AS exhibited potential therapeutic effects. The BF at dose of 400 mg/kg, particularly offered the best anti-inflammatory potential among all treatments (Fig. 10A-C).

The gene expression analysis provides important mechanistic insights into how the *A. santolina* extract and fractions, especially the butanol fraction, were able to protect the brain from the damaging effects of MSG-induced neurotoxicity. The downregulation of pro-inflammatory genes and upregulation of anti-inflammatory genes appears to be a central part of their neuroprotective mode of action.

*A. santolina* methanolic extract (AS), methylene chloride fraction (MF), and butanol fraction (BF) all significantly attenuated the upregulation of the pro-inflammatory genes cox-2 and il-1b that was seen in the MSG-treated group. Conversely, the anti-inflammatory gene il-10 was upregulated in the AS, MF, and BF treatment groups compared to MSG alone.

This modulation of key inflammatory gene targets provides a mechanism by which the *A. santolina* extracts and fractions exerted their observed neuroprotective effects. The downregulation of pro-inflammatory genes like cox-2 and il-1b, coupled with the upregulation of the anti-inflammatory il-10, suggests the extracts and fractions were able to shift the brain’s inflammatory balance away from a detrimental pro-inflammatory state induced by the MSG harm^56^.

Interestingly, the butanol fraction (BF) showed the most potent effects on these inflammatory gene markers. It consistently demonstrated the greatest neuroprotective activity. This indicates the bioactive components concentrated in the BF, were particularly effective at modulating the key inflammatory pathways involved in the MSG-induced neurotoxicity model^57^.

The cerebrum and hippocampus showed neuronal damage due to SMG administration as reported in previous studies^7^. Although L-glutamate is a major excitatory neurotransmitter, its excess cause excitotoxicity due to overactivation of ionotropic glutamate receptors. Furthermore, glutamate was reported to induce oxidative stress like our findings which further aggravate neuronal damage^58^. TNF-α, a cytokine of inflammation, was found to be elevated in the brain of rats exposed to SMG. The increase in TNF-α was found to induce neuronal cell damage due to increased calcium influx in cells which in turn upregulates TNF expression. On the other hand, TNF-α exacerbate the toxic effect of glutamate by inducing its production by microglia cells through upregulation neuronal glutaminase^59^. The neuronal damage observed in SMG group was mitigated partially in other treated groups. Also, the TNF expression in neurons decreased by different grades in treated groups. The decrease was most prominent in BF 400 group indicating the positive effect of BF.

### Molecular docking

The *in vitro* anti-inflammatory study revealed that the isolated compounds (C1-C4) induced inhibitory activities against COX-2 and 5-LOX. Thus, it is necessary to determine the interactions of the isolated compounds in a holistic manner with HO-1, 5-LOX, Keap1 and COX-2 active sites in-silico using molecular docking to determine their pharmacological potential. The results are represented in Tables S2-S5. Among the isolated compounds, isovitexin (C3) exhibited the highest affinity towards HO-1, 5-LOX, Keap1 and COX-2 with docking scores **−** 12.49, -12.73, -15.49 and − 14.18 kcal mol^–^1, respectively. Therefore, it was the most suitable candidate for further molecular dynamic study.

### Molecular dynamic and system stability

To forecast the behavior of the extracted chemicals upon binding to the protein’s active site as well as its interaction and stability through simulation, a molecular dynamic simulation was run^60^. To identify interrupted motions and prevent any artifacts during the simulation, system stability must be validated. The stability of the systems was evaluated in this study using Root-Mean-Square Deviation (RMSD) during the simulations. The stability of the systems was evaluated in this study using Root-Mean-Square Deviation (RMSD) during the simulations. The recorded average RMSD values for all frames of systems apo-protein, and isovitexin - HO-1 systems were 1.59 ± 0.28 Å and 1.23 ± 0.17Å, respectively (Fig. S2A), 1.29 ± 0.19Å, and 1.23 ± 0.20Å, for Apo, isovitexin − 5-LOX, respectively (Fig. S3A), 1.94 ± 0.43Å, and 1.40 ± 0.36Å, for Apo, isovitexin - Keap1, respectively (Fig. S4A) .Finally, 1.46 ± 0.22Å, and 1.36 ± 0.25Å, for Apo isovitexin **-** COX-2, respectively (Fig. S5A). These results revealed that the isovitexin bound to protein complex system acquired a relatively more stable conformation than the other studied systems.

Analyzing the structural flexibility of proteins upon ligand binding during MD simulation is critical for investigating residue behavior and its interaction with the ligand^61^. Using the Root-Mean-Square Fluctuation (RMSF) technique, protein residue variations were evaluated to verify the impact of inhibitor binding to the corresponding targets during the simulations. The computed average RMSF values for all frames of systems apo-protein, and isovitexin - HO-1 systems were 1.11 ± 0.51 Å and 0.95 ± 0.38Å, respectively (Fig. S2B), 1.86 ± 0.59Å, and 1.70 ± 0.53Å, for Apo, isovitexin − 5-LOX, respectively (Fig. S3B), 2.56 ± 0.84Å, and 2.03 ± 0.64Å, for Apo, isovitexin - Keap1, respectively (Fig. S4B). Finally, 2.21 ± 0.71Å, and 1.67 ± 0.49Å, for Apo, isovitexin - COX-2, respectively (Fig. S5B). These values suggested that the isovitexin bound to protein complex system has a lower residue fluctuation than the other systems.

ROG was determined to assess the overall system compactness and stability upon ligand binding during MD simulation^62^. The average Rg values were apo-protein, and isovitexin - HO-1 systems were 24.02 ± 0.16 Å and 23.72 ± 0.11Å, respectively (Fig. S2C), 27.83 ± 0.09Å, and 27.69 ± 0.08Å, for Apo, isovitexin − 5-LOX, respectively (Fig. S3C), and 26.74 ± 0.11Å, and 26.69 ± 0.09Å, for Apo, isovitexin - Keap1, respectively, (Fig. S4C). Finally, 24.53 ± 0.1Å, and 24.25 ± 0.07Å, for Apo, isovitexin - COX-2, respectively (Fig. S5C). Regarding the observed manner, isovitexin bound complex has a highly stiff structure against the catalytic binding site of target receptors.

The compactness of the protein’s hydrophobic core was studied by measuring its solvent accessible surface area (SASA). This was completed by determining the protein’s solvent-visible surface area, which is critical for the stability of biomolecules^63^. The average SASA values were apo-protein, and isovitexin - HO-1 systems were 21518.65 Å and 21167.43Å, respectively (Fig. S2D), 19409.5Å, and 18525.85Å, for Apo, isovitexin − 5-LOX, respectively (Fig. S3D) and 16978.31Å, and 16581.57Å, for Apo, isovitexin - Keap1, respectively, (Fig. S4D) .Finally, 19999.18Å, and 19925.65Å, for Apo, isovitexin - COX-2, respectively (Fig. S5D). The results of the RMSD, RMSF, and ROG calculations, in conjunction with the SASA result, verified that the isovitexin complexe system is still present within the target receptors’ catalytic binding site.

### Binding interaction mechanism based on binding free energy calculation

The molecular mechanics energy methodology (MM/GBSA), which combines generalized Born and surface area continuum solvation, is a widely used method for predicting the free binding energies of small molecules to biological macromolecules and may be more trustworthy than docking scores^64^. The binding free energies were calculated using AMBER18’s MM-GBSA software after obtaining snapshots of the systems’ trajectories. Table 2 shows that, except of ΔGsolv, all reported computed energy components have substantial negative values indicating valuable interactions.

**Table 2:** The calculated energy binding for the compound against the catalytic binding site of target receptor.

Energy components (kcal/mol)					
Complex	ΔE_vdW_	ΔE_elec_	ΔG_gas_	ΔG_solv_	ΔG_bind_
**Human Heme Oxygenase-1 (HO-1)**					
Isovitexin - HO-1	-42.13 ± 0.57	-11.41 ± 0.94	-53.55 ± 0.68	23.47 ± 0.24	-29.80 ± 0.77
Human 5-lipoxygenase (5-LOX)					
Isovitexin-5-LOX	-41.73 ± 0.87	-23.00 ± 0.71	-64.73 ± 0.79	35.99 ± 0.54	-28.74 ± 0.81
**Kelch-like ECH-associated protein (Keap1)**					
Isovitexin - Keap1	-52.01 ± 0.65	-28.23 ± 1.09	-80.24 ± 0.33	41.91 ± 0.68	-38.33 ± 0.82
**Cyclooxygenase-2 (COX-2)**					
Isovitexin - COX-2	-53.68 ± 0.60	-7.35 ± 1.15	-61.03 ± 1.15	20.86 ± 0.90	-40.17 ± 0.62

∆EvdW = van der Waals energy; ∆Eele = electrostatic energy; ∆Gsolv = solvation free energy; ∆Gbind = calculated total binding free energy.

The interactions between the isovitexin compound and the target protein receptor residues are driven by the more positive Vander waals energy component, as proven by a careful investigation of each individual energy contribution, resulting in the stated binding free energies. (Table 2).

### Identification of the critical residues responsible for ligands binding

**Fig. 11:**
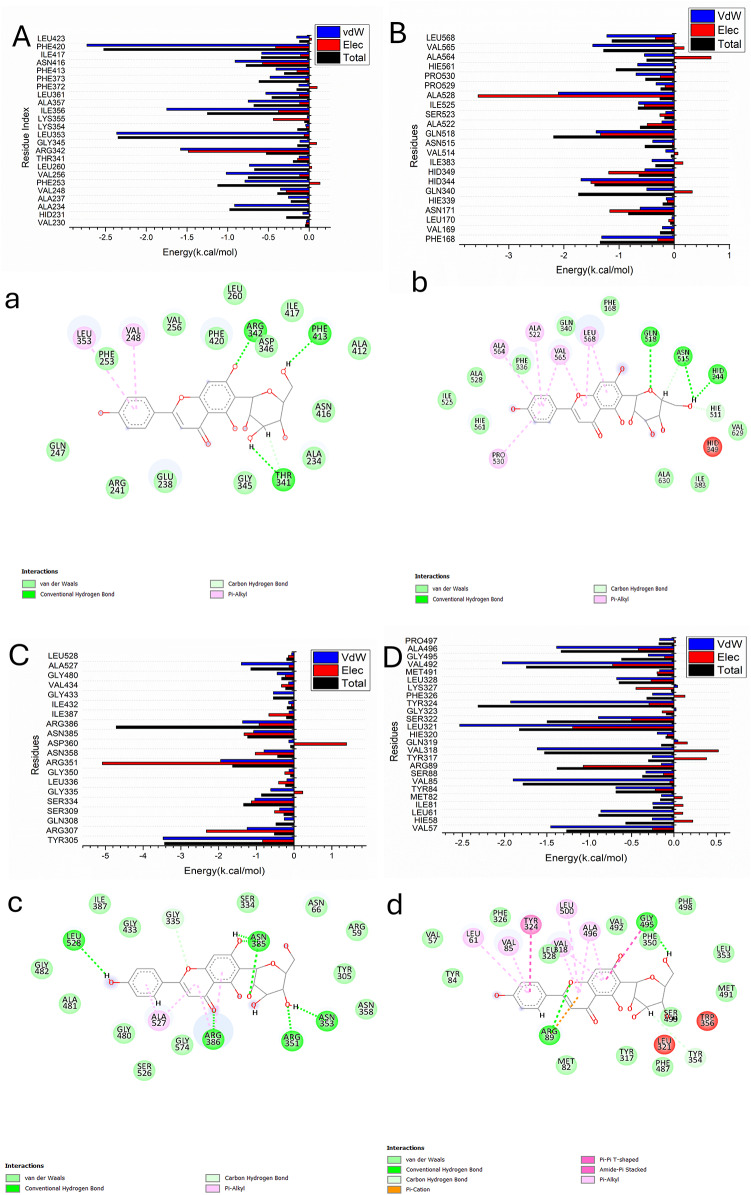
Per-residue decomposition plots showing the energy contributions to the binding and stabilization of isovitexin into catalytic binding site of Human Heme Oxygenase-1 (HO-1) A), human 5-lipoxygenase (5-LOX) B), Kelch-like ECH-associated protein (Keap1) C), and cyclooxygenase-2 (COX-2) D), Corresponding inter-molecular interactions are shown [a], [b], [c], [d].

To learn more about significant residues engaged in the suppression of the catalytic binding site receptor, the total energy involved when these enzymes make chemical interactions was broken down into the role of each site residues. According to Fig. 11, the major favorable contribution of isovitexin compound to the catalytic binding site of Human Heme Oxygenase-1 (HO-1) receptor is mainly observed from residues Ala 234 (-0.916 kcal/mol), Ala237 (-0.255 kcal/mol), Val 248 (-0.353 kcal/mol), Phe 253 ( -0.789 kcal/mol), Val 256 (-1.018 kcal/mol), Leu260 (-0.734 kcal/mol), Arg342 (-1.58 kcal/mol), Leu353 (-2.364 kcal/mol), Ile356 (-1.749 kcal/mol), Ala537 (-0.749 kcal/mol), Leu 361 (-0.532 kcal/mol), Phe 373 (-0.48 kcal/mol), Phe 413 (0.40 kcal/mol), Asn 416 (-0.91 kcal/mol), Ile 417 (-0.583 kcal/mol), and Phe 420 (-2.729 kcal/mol).

Alternatively, the major favorable contribution of isovitexin compound to the catalytic binding site of human 5-lipoxygenase (5-LOX) receptor is chiefly observed from residues Phe 168 (-1.314 kcal/mol), Val 169 (-0.215 kcal/mol), Asn 171 ( -0.618 kcal/mol), Gln 340 ( -0.497 kcal/mol), Hid 344 ( -1.69 kcal/mol), HID 349 ( -0.532 kcal/mol), Ile 383 (-0.40 kcal/mol), Asn 515 (-0.39 kcal/mol), Ala 518 (-1.416 kcal/mol), Ala 522 (-0.224 kcal/mol), Ile525 (-0.646 kcal/mol), Pro 529 ( -0.328 kcal/mol), Pro 530 ( -0.691 kcal/mol), HIE 561( -0.662 kcal/mol), Ala 564 (-0.541 kcal/mol), Val 565 (-1.477 kcal/mol), and Ile525 (-1.221 kcal/mol).

Furthermore, the major favorable contribution of isovitexin compound to the catalytic binding site of Kelch-like ECH-associated protein (Keap1) receptor is predominantly observed from residues Tyr 305 (-3.468 kcal/mol), Arg 307 (-1.241 kcal/mol), Gln 308 (-0.261 kcal/mol), Ser 309 ( -0.381 kcal/mol), Ser 334 (-1.04 kcal/mol), Gly 335 (-0.618 kcal/mol), Leu 336 (-0.187 kcal/mol), Arg351 (-1.946 kcal/mol), Asn 358 (-0.796 kcal/mol), Asn 385 (-1.07 kcal/mol), Asn386 (-1.365 kcal/mol), Gly 433 (-0.55 kcal/mol), Gly 480 (-0.446 kcal/mol), and Ala 527 (-1.396 kcal/mol).

Finally, the major favorable contribution of isovitexin compound to the catalytic binding site of cyclooxygenase-2 (COX-2) receptor is predominantly observed from residues Val 57 (-1.456 kcal/mol), Hie 58 (-0.256 kcal/mol), Leu 61 (-0.863 kcal/mol), Ile 81 ( -0.249 kcal/mol), Tyr 84 (-0.685 kcal/mol), Val 85 (-1.899 kcal/mol), Ser 88 (-0.332 kcal/mol), Tyr 317 (-0.255 kcal/mol), Val 318 (-1.614 kcal/mol), Leu 321(-2.53 kcal/mol), Ser 322 (-0.892 kcal/mol), Tyr 324 (-1.929 kcal/mol), Phe 326 (-0.253 kcal/mol), Leu 328 (-0.677 kcal/mol), Val 492 (-2.027 kcal/mol), Gly 495 (-0.302 kcal/mol), Ala 496 (-1.385 kcal/mol), and Pro 497(-0.169 kcal/mol).

### *In silico* drug-likeness predictions

Tables 3 and 4 present the results of the assessment of the pharmacokinetic properties of the produced ligand. Because pharmacokinetic properties are predominantly responsible for therapeutic utilization, pharmacokinetic evaluation is considered the first screening step for developed drugs. molecules fit the logP and surface area ranges, the number of hydrogen donors and acceptors, and have a molecular weight of 500 g/mol or below. This entails following the Lipinski rule and the oral drugability property of the intended ligands.

LogP values less than 5 for every chemical in Table 3 indicate good absorption and penetration through cell membranes^65^. The number of bond acceptors (NHA) and donors (NHD) in the entire compound is determined by applying the rule of five, as suggested by Lipinski et al. (Table 4). Based on the “LogS prediction” of ~ 2.26 to ~ 2.78, all of the compounds were found to be ascetically soluble, and their synthetic accessibility (3.02–5.29) was within the easy synthetic accessibility range. All of the compounds, interestingly, did not vary from “the Lipinski rule of five,” indicating that each ligand might be used as a lead molecule in a future medicinal endeavor.

**Table 3:** Physicochemical properties and toxicity risks of compounds 3a-5e predicted using DATA warrior.

Compound	Formula	MW	cLogP	clogS	Mutagenic	Tumorigenic	Reproductive effective	Irritant
Isovitexin	C_21_H_20_O_10_	432.38	0.078	-2.269	None	None	None	None
Kaempferol	C_15_H_10_O_6_	286.24	1.8359	-2.787	high	None	None	None
Kaempferol-3-O-glucoside	C_21_H_20_O_11_	448.38	-0.0011	-2.487	None	None	None	None
Luteolin	C_15_H_10_O_6_	286.24	1.99	-2.58	None	None	None	None

**Table 4:** ADME prediction of compounds 3a-5e predicted by Swiss ADME.

Compound	NHD	NHA	NRB	TPSA(A°)	Log *P* (iLogP)	Log S (E SOL)	Synthetic Accessibility
Isovitexin	7	10	3	181.05	1.70	-3.31	4.14
Kaempferol	4	6	1	111.13	1.70	-3.31	3.14
Kaempferol-3-O-glucoside	7	11	4	190.28	1.29	-3.18	5.29
Luteolin	4	6	1	111.13	1.86	-3.71	3.02

## Conclusion

This study revealed the promising efficacy of *A. santolina* extract, fractions, and isolated compounds as antioxidant, anti-inflammatory, and neuroprotective therapies, which is clearly related to their chemical profile and supported by *in vitro*, *in vivo*, and *in silico* studies. The extract and fractions attenuated histopathological alterations in cerebral cortex against glutamate-induced neurotoxicity as well as downregulation of cox-2, IL-1B, and IL-10 gene expression. The isolated compound, isovitexin, showed the highest affinity for HO-1, 5-LOX, Keap1, and COX-2 *in silico*. This highlighted the ethnopharmacological importance of *A. santolina* as an herb used in traditional medicine treating neurological disorders. Therefore, in the sense of developing multi-targeted medications that combine several pharmacological activities having neuroprotective action, *A. santolina* and its constituents are regarded as useful candidates.

## Electronic supplementary material

Below is the link to the electronic supplementary material.

Supplementary Material 1

## Data Availability

All data generated or analyzed during this study are included in this published article and its supplementary information file.

## Abbreviations

ABTS: 2,2’-Azino-bis(3-ethylbenzthiazoline-6-sulfonic acid)
AS: *Achillea santolina* methanolic extract
BF: Butanol fraction
COX-2: Cyclooxygenase-2
DW: Dried weight
FRAP: Ferric reducing antioxidant power
GAE: Gallic acid equivalent
HO-1: Human Heme Oxygenase-1
HPLC: High performance liquid chromatography
IC_50_: Half maximal inhibitory concentration
Keap1: Kelch-like ECH-associated protein
5-LOX: 5-Lipoxygenase
MF: Methylene chloride fraction
QE: Quercetin equivalent
TE: Trolox equivalent
TFC: Total flavonoid content
TPC: Total phenolic content
