# Supplementary material for: Phytochemical analysis and neuroprotective potential of Achillea santolina L. fractions
Source: Sci Rep. 2025 May 8;15:16070. doi: 10.1038/s41598-025-98887-z (PMC12062363; doi:10.1038/s41598-025-98887-z)
Supplement: Supplementary file 1 — Supplementary Material 1 [file 41598_2025_98887_MOESM1_ESM.docx]

**Phytochemical analysis and neuroprotective potential of *Achillea santolina* L. fractions**

**Passent M. Abdel-Baki^1,^*^,^**†**, Nariman E. Mahdy^1,^**†**, Rana M. Ibrahim^1^, Shymaa A.El Badawy^2^, Sara E. Ali^3^, Marwa A. Ibrahim^4^, Marwa S. Khattab^5^, Ahmed A. El-Rashedy^6,7^ , Shimaa R. Emam^2^.**

**^1^**Department of Pharmacognosy, Faculty of Pharmacy, Cairo University, Kasr-El-Ainy Street, Cairo, 11562, Egypt

^2^Department of Pharmacology, Faculty of Veterinary Medicine, Cairo University, Giza, 12211, Egypt. ^3^Department of Physiology, Faculty of Veterinary Medicine, Cairo University, Giza, 12211, Egypt. ^4^Department of Biochemistry and molecular biology, Faculty of Veterinary Medicine, Cairo University, Giza, 12211, Egypt.

^5^Department of Pathology, Faculty of Veterinary Medicine, Cairo University, Giza, 12211, Egypt

^6^Chemistry of Natural and Microbial Products Department, National Research Center (NRC), Dokki 12622, Giza, Egypt.

^7^Department Organic and Medicinal Chemistry, Faculty of Pharmacy, University of Sadat City, Menoufia, 32897, Egypt

*****Corresponding author: Passent M. Abdel-Baki, [passent.mohamed@pharma.cu.edu.eg](mailto:passent.mohamed@pharma.cu.edu.eg)

† These authors contributed equally to this work.

**Material and methods**

**Chemicals**

MSG (≥99%, cas no. 142-47-2) was obtained from Sigma-Aldrich Co. (St. Louis, MO, USA). For chromatography. All the chemicals and reagents were analytical or HPLC grade (Merck, Germany).

**General procedures**

The chromatographic columns were performed using Silica gel H 60, silica gel RP-18, polyamide and sephadex LH-20 (Pharmacia, Stockholm, Sweden). Precoated silica gel 60 F_254_ plates (Sigma-Aldrich Chemicals, Germany) were used for thin layer chromatography (TLC) adopting solvent systems S_1_: methylene chloride-methanol-formic acid (90:10:0.2 *v/v/v*) and S_2_: *n*-butanol-acetic acid-water (4:1:2 *v/v/v*, upper phase). Spots visualization was performed under UV light before and after ammonia vapour exposure and sraying with aluminium chloride or spraying with *p*-anisaldehyde-sulphuric acid, and heating at 110 °C. On Whatmann No. 1 filter paper (Whatmann, Ltd., England), paper chromatography was carried out using the solvent system S_3_ (benzene, n-butane, pyridine, water (1:5:3:3), and aniline phthalate spray reagent was used to visualize the spots. All the solvents utilized for fractionation and extraction were of analytical grade. The melting points were calculated using an electrothermal 9100 (UK) (uncorrected). To record UV spectra, a Jenway model 6800 spectrophotometer was used. The absorbances were measured using a Tecan, microplate reader (Infinite F50, Switzerland). For ^1^H-NMR (400 MHz) and ^13^C-NMR (100 MHz), a Bruker NMR equipment was employed. The NMR spectra in DMSO-d6 were measured. Chemical shifts are expressed in terms of the internal standard TMS and expressed in ppm.

**Preparation and fractionation of *A. santolina* methanolic extract (AS) and fractions (MF and BF)**

*A. santolina* flowering aerial parts (2 kg) were air-dried, powdered, then extracted with methanol (5 L x 4) till exhaustion at room temperature. The combined extracts were subjected to evaporation under reduced pressure at a temperature not exceeding 50 °C, yielding 98 g of dry residue. Two solvents (1 L x 8, each) of various polarities were used to partition the residue (55 g) in a suspension (in 300 mL of distilled water): methylene chloride and *n*-butanol saturated with water. The solvents were concentrated, yielding methylene chloride (MF) and butanol (BF) fractions weighing 18 and 22 g, respectively.

**Phytochemical assessment of *A. santolina* methanolic extract, methylene chloride (MF) and butanol (BF) fractions**

*Total phenolic (TPC) and total flavonoid (TFC) contents determination*

The Folin-Ciocalteu method, as per the earlier reported process ^1^**,** was used to calculate the TPC as gallic acid equivalent (GAE)/mg dried weight (DW). While AlCl_3_ method was used to determine the TFC as quercetin equivalent (QE)/mg DW ^2^**.**

*In vitro antioxidant activity*

Three in vitro assays viz the 2,2`-azino-bis(3-ethylbenzothiazoline-6-sulfonic acid) (ABTS), ferric reducing antioxidant power (FRAP) and oxygen radical absorbance capacity (ORAC) were carried out in accordance with methods previously described ^3,4^ relative to ascorbic acid as a reference antioxidant. The results were given in terms of micromolar (μM) Trolox equivalent (TE) per gram of tested sample (i.e., μM TE/g).

*HPLC analysis of A. santolina methanolic extract (AS)*

HPLC analysis was conducted on Agilent 1260 infinity HPLC Series (Agilent, USA) with a quaternary pump, aKinetex®5µm EVO C18 100 mm x 4.6 mm column (Phenomenex, USA), operated at 30 °C, and a ternary linear elution gradient with (A) HPLC grade water 0.2% H_3_PO_4_ (v/v), (B) methanol, and (C) acetonitrile at a flow rate of 0.2 mL/min. At λ 280 nm, a variable wavelength detector (VWD) was used (Agilent Application Note, Publication number 5991-3801EN, 2014). Three biological replicates were examined. Qualitative determination was accomplished by comparing peak retention times to those of the standard phenolics. While peak area measurement allowed for quantitative determination.

**Isolation of the major phenolics from the methylene chloride fraction (MF)**

The methylene chloride fraction (10 g) was chromatographed on silica gel H 60 (250 g) using a vacuum liquid chromatography column (VLC) (10 L ҳ 12.5 D cm). Fractionation was achieved by adopting gradient elution, beginning with methylene chloride and increasing the polarity with ethyl acetate by 20% until 100% ethyl acetate, and then methanol by 10% increments until 100% pure methanol. Using solvent systems (S_1_-S_2_), 100 mL-sized fractions were collected and TLC-monitored on precoated silica gel plates. Four fractions (I–IV) were obtained by pooling fractions having comparable chromatographic patterns. Fraction III (3.5 g), eluted with 100% ethyl acetate to 10% ethyl acetate in methanol showed two major spots (R*_f_* = 0.5 and 0.55, S_1_) as demonstrated in TLC. Thus, it was chromatographed on a VLC silica gel RP-18 column (20 L X 3 D cm) using methanol: water for elution with stepwise 10% increments of methanol for the isolation and purification of its major compounds. The sub-fraction III-B (1.02 g) eluted with 30% MeOH in H_2_O, showed a single spot that appeared yellow under UV and changed to intense yellow after spraying with aluminium chloride (2%). The solvent was evaporated under reduced pressure and recrystallized with methanol yielding compound C1 **(**yellow crystals, 120 mg, R*_f_* = 0.5, S_1_). The sub-fraction III-C eluted with 40% MeOH in H_2_O (0.9 g), displayed one major spot. It was re-chromatographed on a silica gel 60 column (25 L x 2 D cm) using *n*-hexane: ethyl acetate (80:20 v/v) as solvent system to isolate compound C2 (yellow crystals, 108 mg, R*_f_* = 0.55, S_1_).

**Isolation of the major phenolics from the butanol fraction (BF)**

Fractionation of the butanol fraction (BF) (15 g) was performed using polyamide column (25 L x 5 D cm) adopting gradient elution with 0–100% MeOH in water. Four fractions (I-VI) were collected. Fraction II (2.7 g), eluted with 10-30% MeOH in H_2_O was subjected to chromatography on sephadex LH-20 column (3 D X 35 L cm) using methanol: water (1:1 v/v) for elution. Subfraction II-B (0.67 g) showed a single major deep purple spot under UV light, and when subjected to ammonia and AlCl_3_, it turned yellow. Its purification was achieved by re-chromatography on RP-18 column (1 D x 10 L cm) using water-methanol mixtures yielding compound **C3** (yellow crystals, 102 mg, R*_f_* =0.17, S_2_). Fraction III (1.3 g), eluted with 40-60% MeOH in H_2_O was loaded on sephadex LH-20 column (3 D X 30 L cm) using 50% methanol in water for elution. Subfraction III-B (0.77 g) was purified over sephadex LH-20 column (1.5 D x 13 cm L) adopting MeOH: H_2_O (1:1 v/v) for elution, yielding compound **C4** (yellow crystals, 115 mg, R*_f_* = 0.25, S_2_). Under UV light, compound C4 appeared as a deep purple spot that turned yellow when exposed to ammonia and AlCl_3_.

**Acid hydrolysis for O-glycoside and ferric chloride oxidative hydrolysis of C-glycoside**

The isolated compounds (C3 and C4) were subjected to acid hydrolysis (2 N HCl, 2 h, 100^◦^C) followed by co-paper chromatography (PC) with authentic sugar moieties. Compound C4 was subjected to oxidative hydrolysis using ferric chloride (20% FeCl_3_, 6 h) followed by co-PC against standard sugars using solvent system S_3_ ^5^.

**Data of the isolated compounds from the methylene chloride fraction (MF)**

*Luteolin (C1)*

Yellow crystals; melting point: 237-237°C; *Rf.* 0.5 (S_1_). UV spectral data, λ_max_ (nm): MeOH: 256sh, 267, 345; MeOH/NaOMe: 274, 321sh, 409; AlCl_3_: 272, 300, 361, 399; AlCl_3_/HCl: 272, 255, 388; NaOAc: 273, 343, 401; NaOAc/H_3_BO_3_: 260, 371. ^1^H-NMR (400 MHz, DMSO-*d_6_*): δ (ppm); 7.2541 (1H, dd, *J* = 2.0, 8.5 Hz, H-6′),7.2198 (1H, d, *J* = 2.0 Hz, H-2′), 6.5441 (1H, d, *J* = 8.5 Hz, H-5′), 6.3712 (1H, s, H-3), 6.1512 (1H, d, *J* = 2.0Hz, H-8), 5.9111 (1H, d, *J* = 2.0Hz, H-6).^13^C-NMR (100 MHz, DMSO-*d_6_*): δ (ppm); 95.1 (C-8), 100.2 (C-6), 103.8 (C-3), 105.4 (C-10), 114.2 (C-2′), 116.9 (C-5′), 120.4 (C-6′), 123.9 (C-1′), 147.1 (C-3′), 151.1 (C-4′), 159.5 (C-9), 163.1 (C-5), 166.0 (C-2), 166.5 (C-7), 183.8 (C-4).

*Kaempferol (C2)*

Yellow crystals; melting point: 276-277°C; *Rf =* 0.55 (S_1_). UV spectral data, λ_max_ (nm):MeOH: 267,293sh, 320sh, 367; NaOMe: 277, 321sh, 412; AlCl_3_: 270, 306, 351, 424; AlCl_3_/HCl: 268, 306, 351, 425; NaOAc: 275, 307, 379; NaOAc/H_3_BO_3_: 267, 296, 321, 370.^1^H NMR (400 MHz, DMSO-*d6*): δ (ppm); 8.0411 (2H, d, *J* = 8.5, H-2',6'); 6.9299 (2H,d, *J* = 8.5, H-3',5'); 6.4121 (1H, d, *J* = 2.0, H-8); 6.1616 (1H,d, *J* = 2.0, H-6). ^13^C-NMR (100 MHz, DMSO-*d_6_*): δ (ppm); 93.5 (C-8), 98.2 (C-6), 103.1 (C-10), 115.5 (C-3′, C-5′), 121.9 (C-1′), 129.6 (C-2′- C-6′), 135.8 (C-3), 146.9 (C-2), 156.1 (C-9), 159.1 (C-4′), 160.6 (C-5), 163.8 (C-7), 176.0 (C-4).

**Data of the isolated compounds from the butanol fraction (BF)**

*Apigenin 6-C-β-glucopyranoside (isovitexin) (C3)*

Yellow crystals, *Rf.* 0.17 (BAW). UV spectral data, λ_max_(nm): MeOH: 273, 328; MeOH/NaOMe: 284, 326, 395; AlCl_3_: 277, 304, 348; AlCl_3_/HCl: 277, 301, 342; NaOAc: 281, 384; NaOAc/H_3_BO_3_: 276, 317, 357. ^1^H-NMR (400 MHz in DMSO-*d_6_,* δ, ppm, *J*/Hz): δ 7.9691 (2H, d, *J* = 8.6 Hz, H-2′, H-6′), 6.8441 (2H, d, *J* = 8.6 Hz, H-3',5′), 6.6313 (1H, s, H-3), 6.4212 (1H, s, H-8), 4.5392 (1H, d, *J* = 9.6 Hz, H-1′′), 3-4 (10H, m, H-2′′- H-6′′). ^13^C NMR (100 MHz, DMSO-*d_6_,* δ, ppm): 182.5 (C-4), 162.2 (C-2), 161.6 (C-5, C-7), 161.2 (C-4' ), 155.4 (C-9), 128.9 (C-2'), 121.6 (C-10 ), 115.1 (C-3' ,5' ), 104.2 (C-6), 103.7 (C-10), 101.2 (C-3), 97.4 (C-8), 81.3 (C-5''), 80.2 (C-3''), 72.6 (C-1''), 70.1 (C-2''), 70.3 (C-4''), 61.5 (C-6'').

*Kaempferol 3-O-β-glucopyranoside (astragalin) (****C4****)*

Yellow crystals, *Rf.* 0.25 (S_2_). UV spectral data, λ_max_ (nm): MeOH: 264, 344; MeOH/NaOMe: 272, 325, 400; AlCl_3_: 272, 302, 347, 394; AlCl_3_/HCl: 272, 343, 392; NaOAc: 272, 303, 391; NaOAc/H_3_BO_3_: 265, 350. ^1^H-NMR (400 MHz in DMSO-*d_6_*, δ, ppm, *J*/Hz): δ 7.8212 (2H, d, *J* = 8.5 Hz, H-2′, H-6′), 6.7811 (2H, d, *J* = 8.5 Hz, H-3',5′), 6.3522 (1H, d, *J* = 2.0 Hz, H-8), 6.1411 (1H, d, *J* = 2.0 Hz, H-6), 5.3310 (1H, d, *J* = 7.5 Hz, H-1′′), 3-4 (5H, m, H-2′′- H-6′′, H-2′′). ^13^C NMR (100 MHz, DMSO-*d_6_,* δ, ppm): 177.5 (C-4), 164.4 (C-7), 161.2 (C-5), 159.9 (C-4' ), 156.4 (C-2), 156.2 (C-9 ), 133.1 (C-3), 130.9 (C-2' ,6' ), 120.9 (C-1'), 115.1 (C-3', 5'), 103.9 (C-10), 100.8 (C-1''), 98.7 (C-6), 93.7 (C-8), 77.6 (C-5''), 76.4 (C-3''), 74.3 (C-2''), 69.9 (C-4''), 60.7 (C-6'').

**In vitro anti-inflammatory activity of the isolated compounds (C1-C4)**

*In vitro cyclooxygenase (COX-2) inhibitory activity*

In a 96-well plate, serial dilutions of the isolated compounds and standard (250-0.98 µM) were done. The inhibitory activity of the tested samples against COX-2 enzyme was determined colorimetrically ^6^. Celecoxib was used as a reference drug. The IC_50_ (concentration at which the tested sample produces 50% inhibition) of COX-2 was calculated.

*In vitro lipoxygenase (5-LOX) inhibitory activity*

The 5-LOX inhibitory activity was determined according to a previous method ^7^. A Serial dilution of each tested compounds and standard (125- 0.98 µM) was prepared in DMSO in a 96-well plate. Zileuton was used as a reference drug. The IC_50_ of each tested sample was calculated.

## **System preparation and Molecular Docking**

The crystal structures of Human Heme Oxygenase-1 (HO-1), Human 5-lipoxygenase (5-LOX), Kelch-like ECH-associated protein (Keap1), and Cyclooxygenase-2 (COX-2) were retrieved from the protein data bank with code 3HOK ^8^, 3V99 ^9^, 4L7B ^10^, 6COX ^11^ and prepared using UCSF Chimera ^12^. Using PROPKA, pH was fixed and optimized to 7.5^13^. Extracted 2D structure was drawn using ChemBioDraw Ultra 12.1^14^. The steepest descent approach and MMFF94 force field in Avogadro software ^15^ were used to optimise 2D structure for energy minimization. In preparation for docking, hydrogen atoms were removed using UCSF chimaera ^12^.

## **Molecular docking**

The structures of the extracted compounds were drawn using ChemOffice tool (ChemDraw 16.0) assigned with proper 2D orientation^14^ . The energy of each molecule was minimized using ChemBio3D and were then used as input for AutoDock Vina, in order to carry out the docking simulation. The protein preparation was done using the reported standard protocol by removing the co-crystallized ligand, water molecules , and cofactors; the target protein file was prepared by leaving the associated residue with protein using Auto preparation of target protein file AutoDock 4.2 ^16^ (MGLTools 1.5.6) ^18^. The graphical user interface program was used to set the grid box for docking simulations. The grid was set so that it surrounds the region of interest in the macromolecule. The docking algorithm provided with AutoDock Vina v.1.2.0 was used to search for the best docked conformation between ligand and protein. 18.31, -2.06, 30.83 for 3HOK; 10.75, -79.23, -39.40 for 3V99; 2.78, -1.11, -29.57 for 4L7B; and 24.44, 21.59, 45.69 for 6COX are the x, y, and z AutoDock Vina grid center coordinates that were used. In all three situations, the search space's dimensions were set to 20 Å x 20 Å x 20 Å, with an exhaustiveness value of 8. During the docking process, a maximum of nine conformers were considered for each ligand. Then, the resulting docking poses were visually examined with BIOVIA Discovery Studio, and interactions with binding pocket residues were studied. Poses fitting into the binding pocket with the top scores and showing useful ligand enzyme contacts were selected.

## **Molecular dynamic (MD) simulations**

The incorporation of Molecular Dynamic (MD) simulations into the study of biological systems allows for the exploration of atoms and molecules' physical motion, which is not easily accessible by other means ^20^. The information collected from conducting this simulation provides a complete insight of the dynamical evolution of biological systems, including molecular affiliation and conformational changes ^20^. The AMBER 18 package included the PMEMD GPU engine, was utilized to run the MD simulations for every system ^21^. The partial atomic charge of each compound was calculated using ANTECHAMBER's General Amber Force Field (GAFF) technique ^22^. The AMBER 18 package's Leap module implicitly solvated each system within 10 Å of each box edge within an orthorhombic box containing TIP3P water molecules. Na^+^ and Cl^-^ counter ions were added to each system using the Leap module to neutralize it. Each system experienced a 2000-step initial minimization with an imposed restraint potential of 500 kcal/mol, followed by a 1000-step full minimization using the conjugate gradient algorithm in the absence of constraints.

To guarantee that every system had the same number of atoms and volume throughout the MD simulation, each system was gradually heated over 500ps, from 0K to 300K. The collision frequency for the solutes in the system was 1 ps, with a possible harmonic limitation of 10 kcal/mol. Every system was heated to a constant temperature of 300K and allowed to equilibrate for 500ps. To represent an isobaric-isothermal (NPT) ensemble, the number of atoms and pressure in each system were kept constant throughout the production simulation. The system pressure was kept at 1 bar using a Berendsen barostat ^23^.

Every system underwent MD simulation for 20 ns. In each simulation, the hydrogen bond atoms were constrained using the SHAKE technique. Every simulation integrated an SPFP precision model and used a 2fs step size. Simulations were conducted using an isobaric-isothermal ensemble (NPT) with randomised seeding, a constant pressure of 1 bar, a pressure-coupling constant of 2ps, a temperature of 300K, and a Langevin thermostat with a collision frequency of 1ps.

**Post-MD Analysis**

## The CPPTRAJ ^24^ module of the AMBER18 suite was used to examine the trajectories, which were saved every 1 ps from the MD simulations. All graphs and visualizations were made using Chimera ^12^ and Origin ^25^, a data analysis tool.

## **Thermodynamic calculation**

The Poisson-Boltzmann or generalised Born and surface area continuum solvation (MM/PBSA and MM/GBSA) technique has been shown to improve ligand-binding affinity calculations ^26^. Within a given force field, the Protein-Ligand complex molecular simulations used by MM/GBSA and MM/PBSA provide precise statistical-mechanical binding free energy. Average binding free energy over 200 images taken from the full 20 ns trajectory. The following representation ^27^ can be used to estimate the change in binding free energy (ΔG) for each molecular species (complex, ligand, and receptor):

$$\Delta G_{\mathrm{bind}}=G_{\mathrm{complex}}-G_{\mathrm{receptor}}-G_{\mathrm{ligand}} \left( 1 \right)$$

$$\Delta G_{\mathrm{bind}}=E_{\mathrm{gas}}+G_{\mathrm{sol}}-TS \left( 2 \right)$$

$$E_{\mathrm{gas}}=E_{\mathrm{int}}+E_{\mathrm{vdw}}+E_{\mathrm{ele}} \left( 3 \right)$$

$$G_{\mathrm{sol}}=G_{\mathrm{GB}}+G_{\mathrm{SA}} \left( 4 \right)$$

$$G_{\mathrm{SA}}=\gamma SASA \left( 5 \right)$$

The terms Egas, Eint, Eele, and Evdw represent the gas-phase energy, internal energy, Coulomb energy, and van der Waals energy, respectively. The FF14SB force field words were utilized to directly evaluate the Egas. The solution-free energy (Gsol) was calculated using the energy involved from the polar and non-polar states (GGB and G, respectively). Using a water probe radius of 1.4 Å, the non-polar solvation free energy (GSA) was computed from the Solvent Accessible Surface Area (SASA) ^28^. On the other hand, the polar solvation (GGB) contribution was calculated by solving the GB equation. Item S represents the solute's total entropy, while item T represents temperature. The contribution of each residue to the total binding free energy was determined using Amber18's MM/GBSA-binding free energy technique.

**Computation of drug-like parameters and ADMET profiling**

*In silico Drug-likeness predictions*

The drug-likeness of a pharmacological agent can be utilized as a criterion to assess whether or not it has properties that would qualify it as an oral active herbicide ^29^. The "Lipinski rule of five," which was created by Lipinski et al. ^30^, provides the foundation for this forecast. Using DATA Warrior and the "Swiss ADME" ^31^ predictor, "in silico" predictions of the herbicide-likeness and toxicity of the investigated ligands were produced. The solubility, cLogP, total polar surface area (TPSA), hydrophilicity (LogP), molecular mass, and potential for mutation, toxicity, irritation, and reproduction of each chemical are all assessed by the DATA Warrior program. Meanwhile, the Swiss ADME predictor provides information about the synthetic accessibility of the compounds and the amount of "hydrogen donors, acceptors, and rotatable bonds".

**Supplementary Figures**

| 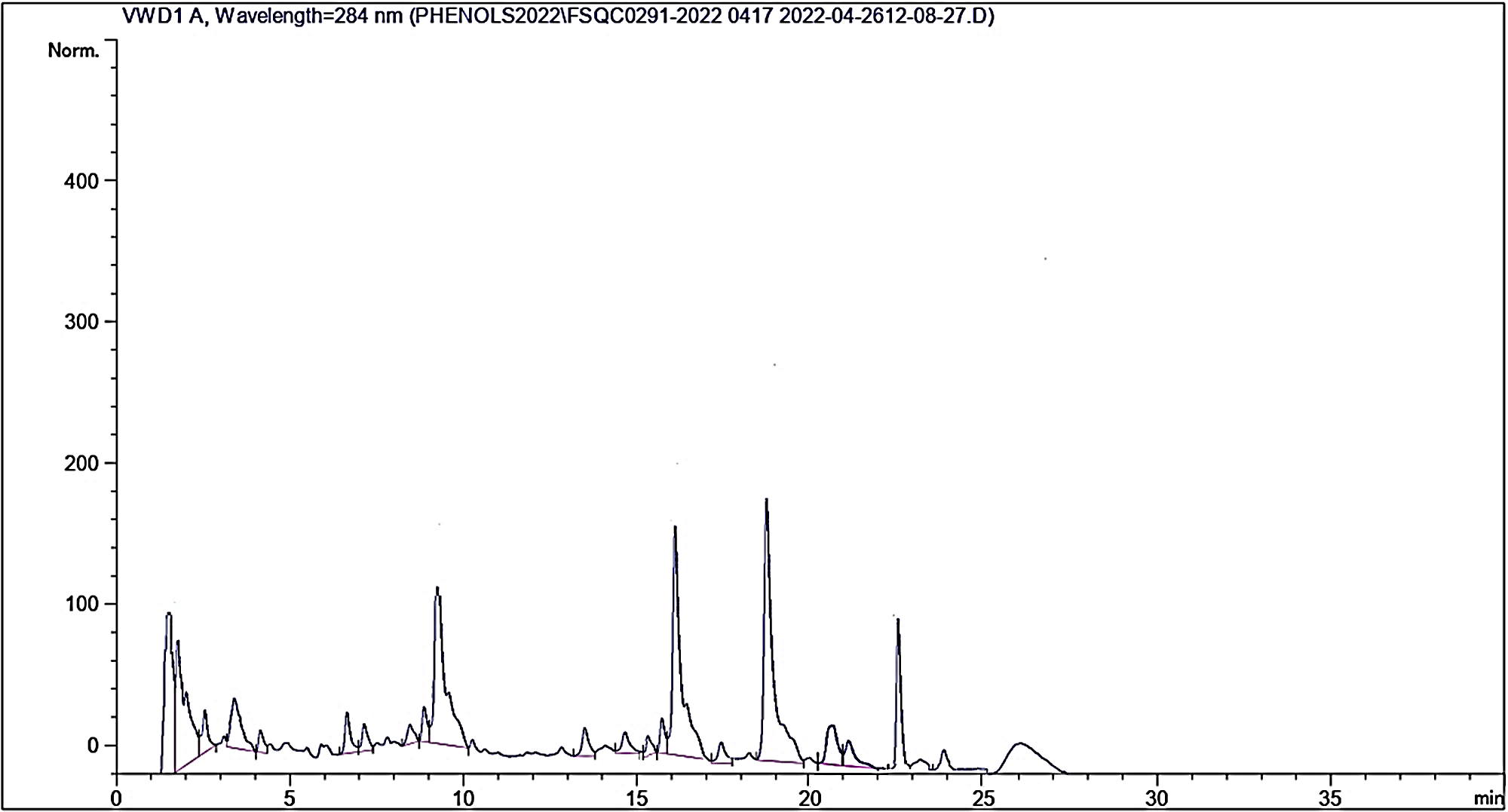 |
| --- |
| Figure S1 HPLC chromatogram showing identified phenolic compounds in the methanolic extract of *A. santolina* (AS) flowering aerial parts measured at 280 nm.  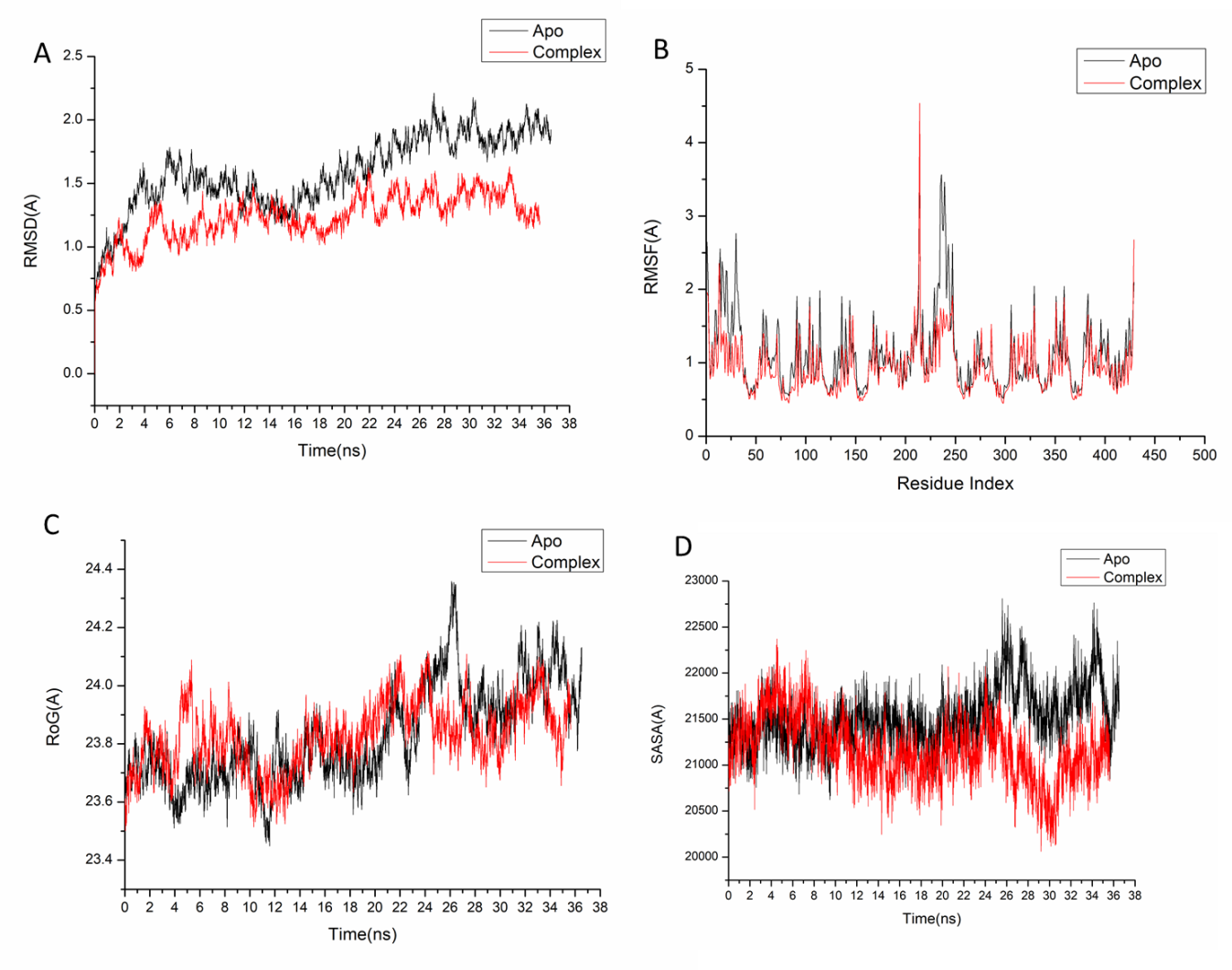  **Figure S2** A) RMSD of Cα atoms of the protein backbone atoms. B) RMSF of each residue of the protein backbone Cα atoms of protein residues C) ROG of Cα atoms of protein residues; D) solvent accessible surface area (SASA) of the C α of the backbone atoms relative (black) to the starting minimized over 38 ns for the catalytic binding site with isovitexin - HO-1 complex system (red).  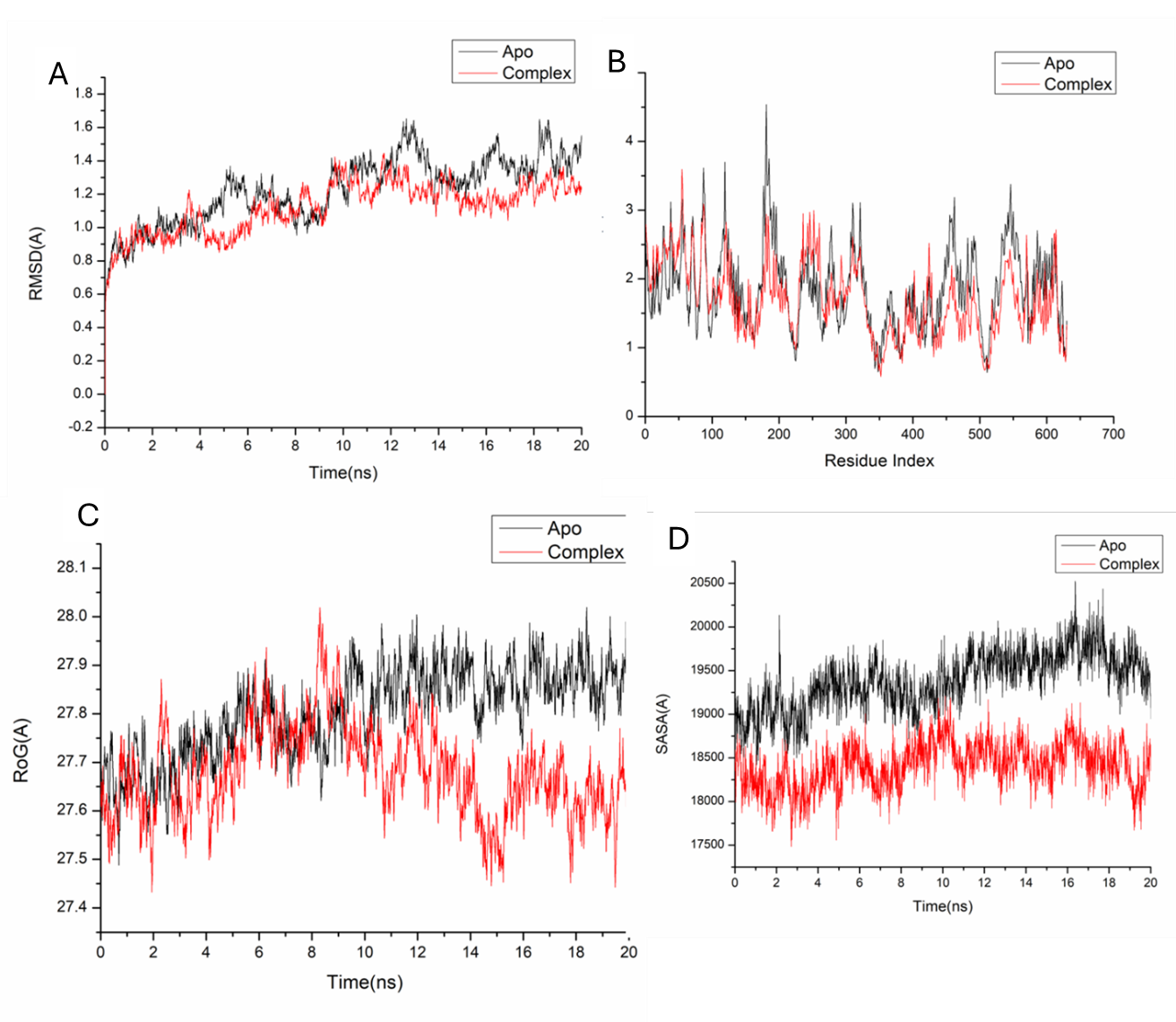  **Figure S3** A) RMSD of Cα atoms of the protein backbone atoms. B) RMSF of each residue of the protein backbone Cα atoms of protein residues C) ROG of Cα atoms of protein residues; D) solvent accessible surface area (SASA) of the C α of the backbone atoms relative (black) to the starting minimized over 20 ns for the catalytic binding site with isovitexin - 5-LOX complex system (red).  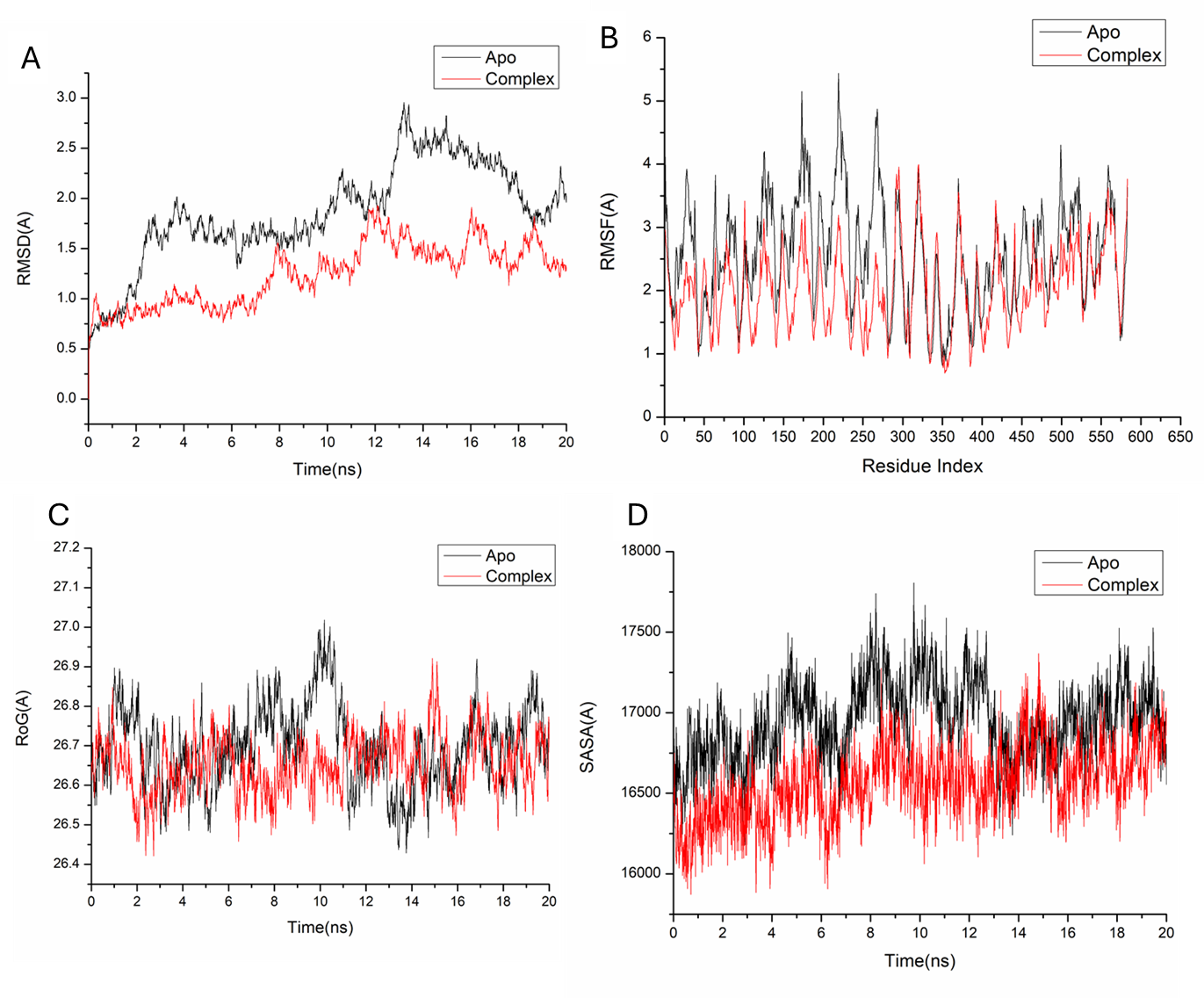  **Figure S4** A) RMSD of Cα atoms of the protein backbone atoms. B) RMSF of each residue of the protein backbone Cα atoms of protein residues C) ROG of Cα atoms of protein residues; D) solvent accessible surface area (SASA) of the C α of the backbone atoms relative (black) to the starting minimized over 20 ns for the catalytic binding site with isovitexin - Keap1 complex system (red).  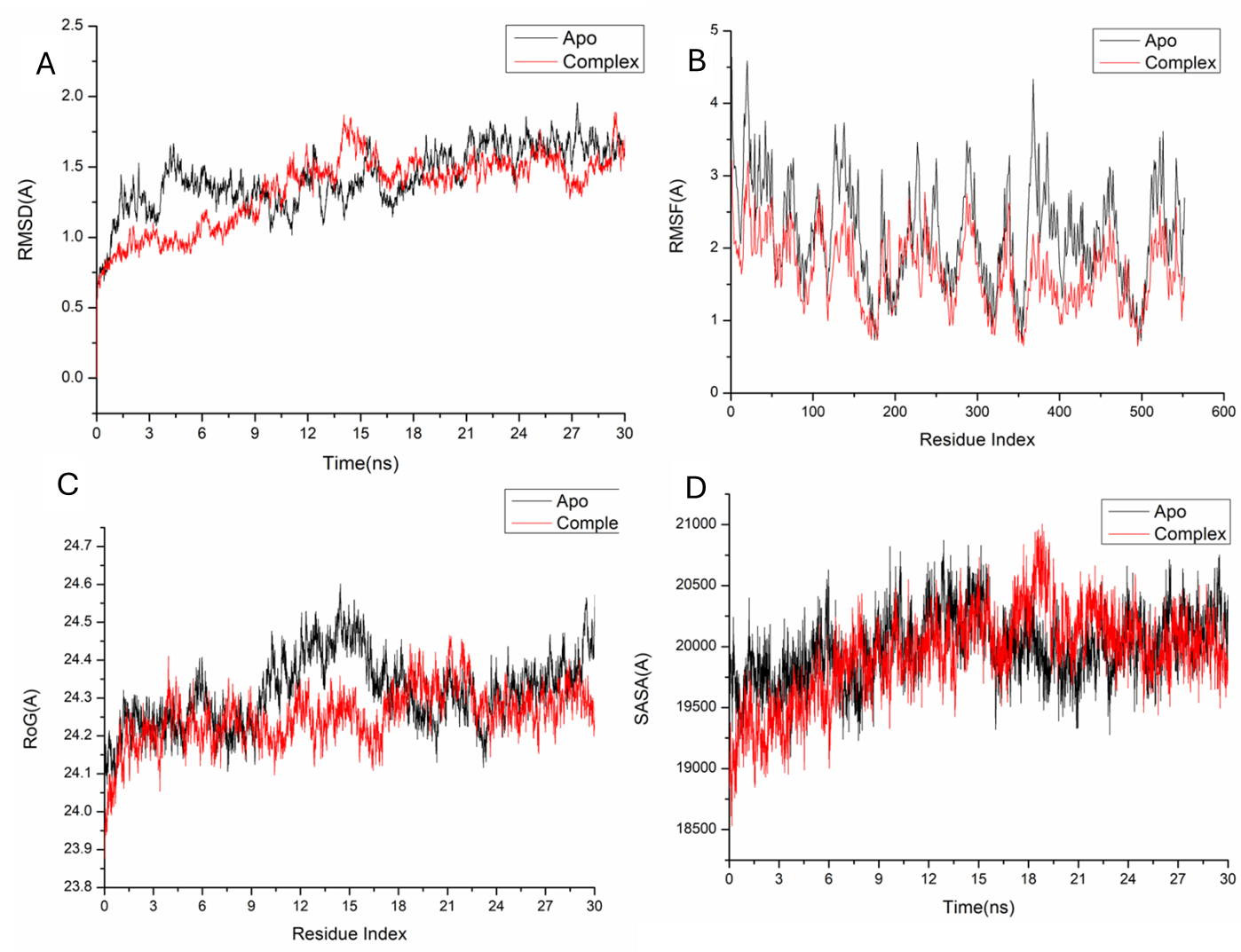  Figure S5 A) RMSD of Cα atoms of the protein backbone atoms. B) RMSF of each residue of the protein backbone Cα atoms of protein residues C) ROG of Cα atoms of protein residues; D) solvent accessible surface area (SASA) of the Cα of the backbone atoms relative (black) to the starting minimized over 30 ns for the catalytic binding site with isovitexin - COX-2 complex system (blue). |

**Supplementary Tables**

**Table S1** Gene primers and accession numbers.

| Gene | Forward primer | Reverse primer | Accession number | Reference |
| --- | --- | --- | --- | --- |
| COX-2 | AGGAGCATCCTGAGTGGGAT | AGAAGCGTTTGCGGTACTCA | L25925.1 | ^32,33^ |
| IL-1β | TTGAGTCTGCACAGTTCCCC | GTCCTGGGGAAGGCATTAGG | [NM_031512.2](https://www.ncbi.nlm.nih.gov/entrez/viewer.fcgi?db=nucleotide&id=158186735) | ^34^ |
| IL-10 | TCCCTGGGAGAGAAGCTGAA | CCTGCAGTCCAGTAGATGCC | [NM_012854.2](https://www.ncbi.nlm.nih.gov/entrez/viewer.fcgi?db=nucleotide&id=148747381) | ^35^ |
| ACTB | CCGCGAGTACAACCTTCTTG | CAGTTGGTGACAATGCCGTG | NM_031144.3 | ^33^ |

**Table S2** Autodocking vina docking results for Extracted compounds docked into the catalytic domain binding site of Human Heme Oxygenase-1 receptor in comparison to the co-crystallized Q80 ligand

| Compounds | Hydrogen bonds between atoms of compounds and amino acids of receptor | | | | | S- score  (binding energy)  (kcal/mol) |
| --- | --- | --- | --- | --- | --- | --- |
|  | **Compounds** | **Receptors** | | **Type** | **Distance (Å)** |  |
|  | **Atoms** | **Atoms** | **Residues** |  |  |  |
| Co crystalized ligand | O7029 | NH5597 | Arg136 | H-acceptor | 4.75 | -10.73 |
| Isovitexin | H7046 | O6705 | Phe 207 | H-donor | 2.15 | -12.49 |
|  | O7029 | ND6769 | Asn210 | H-acceptor | 2.83 |  |
| Kaempferol | H7028 | O5567 | Thr135 | H-donor | 2.61 | -9.28 |
| Kaempferol-3-O-glucoside | H7039 | OD5657 | Asp 140 | H-donor | 2.72 | -10.29 |
| Luteolin | H7029 | OD5657 | Asp 140 | H-donor | 3.27 | -9.80 |
| Gallic acid | No interaction |  |  |  |  |  |
| Protocatechuic acid | H7015 | O3795 | Ala28 | H-donor | 2.39 | -8.96 |
|  | H7014 | O6777 | Ile 211 | H-donor | 2.11 |  |
|  | H7013 | O6832 | Phe214 | H-donor | 2.65 |  |
| Chlorogenic acid | H7039 | O6777 | Ile211 | H-donor | 2.12 | -8.96 |
| Vanillic acid | H7018 | O6777 | Ile211 | H-donor | 2.95 | -7.16 |
| Caffeic acid | H7018 | O6832 | Phe214 | H-donor | 2.04 | -8.12 |
| Ferulic acid | No interaction |  |  |  |  |  |
| Orientin | H7047 | OE3852 | Glu32 | H-donor | 1.99 | -9.77 |
|  | H7049 | OD5657 | Asp140 | H-donor | 2.82 |  |
| vitexin | O7029 | NH3912 | Arg35 | H-acceptor | 2.57 | -9.27 |
| Isoorientin | H7044 | OD5657 | Asp140 | H-donor | 1.28 | -9.60 |
| Rutin | No interaction |  |  |  |  |  |
| Isoquercitrin | No interaction |  |  |  |  |  |
| Isorhamnetin-3-O-glucoside | No interaction |  |  |  |  |  |
| Myricetin | H7029 | O4000 | Gln41 | H-donor | 2.97 | -10.06 |
| Quercetin | H7028 | OE3852 | Glu32 | H-donor | 2.98 | -9.71 |
| Apigenin | H7026 | O3795 | Ala28 | H-donor | 3.20 | -9.32 |
| Kaempferol | H7027 | O6777 | Ile211 | H-donor | 2.49 | -9.36 |
| isorhamnetin | No interaction |  |  |  |  |  |
| Rhamnetin | H7032 | OE3853 | Glu32 | H-donor | 1.52 | -9.46 |

**Table S3** Autodocking vina docking results for Extracted compounds docked into the catalytic domain binding site of human 5-lipoxygenasein comparison to the co-crystallized Arachidonic Acid ligand

| Compounds | Hydrogen bonds between atoms of compounds and amino acids of receptor | | | | | S- score  (binding energy)  (kcal/mol) |
| --- | --- | --- | --- | --- | --- | --- |
|  | **Compounds** | **Receptors** | | **Type** | **Distance**  **(Å)** |  |
|  | **Atoms** | **Atoms** | **Residues** |  |  |  |
| Co crystalized ligand | H10260  H10217 | O10295  O10953 | Val 50  Ser52 | H-donor  H-donor | 1.78  1.99 | -14.06 |
| Isovitexin | H 20544 | O6273 | Lys 409 | H-donor | 1.66 | -12.73 |
|  | H20544 | O6273 | Ala410 | H-donor | 2.52 |  |
| Kaempferol | O20522 | NZ6286 | Lys409 | H-acceptor | 3.08 | -10.11 |
| Kaempferol-3-O-glucoside | O20532 | N2703 | Phe177 | H-donor | 2.50 | -10.71 |
| Luteolin | H20535 | OE6356 | Gln413 | H-donor | 2.05 | -10.58 |
| Gallic acid | H10190 | O9132 | Leu607 | H-donor | 3.24 | -9.26 |
| Protocatechuic acid | O10185 | NZ6286 | Lys409 | H-acceptor | 2.88 | -7.29 |
| Chlorogenic acid | No interaction |  |  |  |  |  |
| Vanillic acid | No interaction |  |  |  |  |  |
| Caffeic acid | No interaction |  |  |  |  |  |
| Ferulic acid | No interaction |  |  |  |  |  |
| Orientin | H10227 | O6295 | Ala410 | H-donor | 3.27 | -6.29 |
| vitexin | H10213 | O2697 | Ser171 | H-donor | 2.08 | -10.24 |
| Isoorientin | H10226 | O8334 | Phe555 | H-donor | 3.11 | -10.58 |
| Rutin | O10212 | OD2769 | Asn180 | H-donor | 3.00 | -8.28 |
| Isoquercitrin | H10225 | OE5511 | Gln363 | H-donor | 3.17 | -10.86 |
| Isorhamnetin-3-O-glucoside | H10219 | O10171 | Ala672 | H-donor | 2.39 | -9.28 |
| Myricetin | H10207 | O6295 | Ala410 | H-donor | 2.43 | -9.25 |
| Quercetin | O10194 | N2703 | Phe177 | H-donor | 2.09 | -8.69 |
| Apigenin | O10193 | OG2701 | Ser171 | H-donor | 2.95 | -7.86 |
| Kaempferol | O10194 | N2703 | Phe177 | H-donor | 2.18 | -8.84 |
| isorhamnetin | H10210 | OE5511 | Gln363 | H-donor | 2.91 | -9.32 |
| Rhamnetin | H10204 | O10171 | Ala672 | H-donor | 2.37 | -8.26 |
|  | O101777 | N2703 | Phe177 | H-donor | 3.05 |  |

**Table S4** Autodocking vina docking results for Extracted compounds docked into the catalytic domain binding site of Kelch-like ECH-associated protein (Keap1)

| Compounds | Hydrogen bonds between atoms of compounds and amino acids of receptor | | | | | S- score  (binding energy)  (kcal/mol) |
| --- | --- | --- | --- | --- | --- | --- |
|  | **Compounds** | **Receptors** | | **Type** | **Distance**  **(Å)** |  |
|  | **Atoms** | **Atoms** | **Residues** |  |  |  |
| Co crystalized ligand | O8822  O8818 | NH5766  OG8624 | Arg415  Ser602 | H-acceptor  H-acceptor | 2.82  2.69 | -10.911 |
| Isovitexin | H8813 | OD5295 | Asn382 | H-donor | 1.39 | -15.49 |
|  | O8783 | OD5744 | Asn414 | H-donor | 3.06 |  |
| Kaempferol | O8782 | OG5046 | SER 363 | H-donor | 2.66 | -11.96 |
| Kaempferol-3-O-glucoside | O8782 | OG5046 | Ser363 | H-don | 1.73 | -13.007 |
| Luteolin | H8783 | N956 | Asn387 | H-acceptor | 2.85 | -12.38 |
| Gallic acid | O8773 | OH4589 | Tyr334 | H-donor | 1.83 | -10.38 |
| Protocatechuic acid | O8773 | OH4589 | Tyr334 | H-donor | 2.71 | -10.13 |
| Chlorogenic acid | O8787 | OG5046 | Ser963 | H-donor | 2.56 | -11.59 |
| Vanillic acid | O8775 | OG5046 | Ser963 | H-donor | 2.91 | -9.73 |
| Caffeic acid | O8776 | ND5745 | Asn414 | H-acceptor | 2.85 | -10.81 |
| Ferulic acid | O8776 | OH4589 | Tyr334 | H-donor | 2.26 | -10.54 |
| Orientin | H8812 | O927 | Pro384 | H-donor | 2.20 | -13.17 |
| vitexin | H8815 | OD5353 | Asn387 | H-donor | 2.64 | -12.62 |
| Isoorientin | O8792 | NH5769 | Arg415 | H-acceptor | 3.19 | -11.91 |
| Rutin | O8793 | OG5046 | Ser363 | H-acceptor | 1.02 | -13.21 |
| Isoquercitrin | O8794 | OG5046 | Ser363 | H-donor | 2.45 | -13.34 |
| Isorhamnetin-3-O-glucoside | O8798 | OG7922 | Ser555 | H-donor | 1.63 | -12.39 |
| Myricetin | O8786 | OG8624 | Ser602 | H-donor | 2.09 | -13.58 |
| Quercetin | O8785 | OG5982 | Ser431 | H-donor | 2.76 | -12.82 |
| Apigenin | O8784 | OH8182 | Tyr 572 | H-donor | 1.86 | -11.33 |
| Kaempferol | O8782 | OH4589 | Tyr334 | H-donor | 2.57 | -12.21 |
| isorhamnetin | O8784 | OG8624 | Ser602 | H-donor | 2.25 | -12.22 |
|  | H8796 | O8620 | Ser602 | H-donor | 1.51 |  |
| Rhamnetin | O8777 | OG5046 | Ser363 | H-acceptor | 3.00 | -12.23 |
|  | O8785 | NH878 | Arg380 | H-acceptor | 2.43 |  |

**Table S5** Autodocking vina docking results for Extracted compounds docked into the catalytic domain binding site of cyclooxygenase-2 (COX-2) in comparison to the co-crystallized SC-558 ligand

| Compounds | Hydrogen bonds between atoms of compounds and amino acids of receptor | | | | | S- score  (binding energy)  (kcal/mol) |
| --- | --- | --- | --- | --- | --- | --- |
|  | **Compounds** | **Receptors** | | **Type** | **Distance**  **(Å)** |  |
|  | **Atoms** | **Atoms** | **Residues** |  |  |  |
| Co crystalized ligand | H8976  H8975 | OE1294  O2605 | Gln192  Leu352 | H-donor  H-donor | 2.71  3.43 | -14.34 |
| Isovitexin | O17758 | OH5736 | Tyr385 | H-donor | 2.60 | -14.18 |
|  | H17775 | O8004 | Gly526 | H-donor | 2.52 |  |
| Kaempferol | O17746 | OG8058 | Ser530 | H-donor | 2.68 | -12.74 |
| Kaempferol-3-O-glucoside | H17776 | O5112 | Val349 | H-donor | 3.17 | -12.30 |
| Luteolin | H17756 | OE2561 | Gln192 | H-donor | 3.56 | -12.98 |
| Gallic acid | O8873 | OH5736 | Tyr385 | H-donor | 1.82 | -10.22 |
| Protocatechuic acid | O8871 | OH5736 | Tyr385 | H-donor | 2.46 | -9.26 |
| Chlorogenic acid | H8896 | O7936 | Met522 | H-donor | 1.86 | -10.27 |
|  | H8895 | O7936 | Gly526 | H-donor | 1.52 |  |
| Vanillic acid | No interaction |  |  |  |  |  |
| Caffeic acid | H8881 | OE2561 | Gln192 | H-donor | 2.50 | -10.30 |
| Ferulic acid | O8875 | OG8085 | SER530 | H-donor | 3.03 | -10.14 |
| Orientin | O8890 | OH5736 | Tyr385 | H-donor | 2.72 | -11.52 |
| vitexin | O8889 | OG8058 | Ser530 | H-donor | 1.69 | -11.67 |
| Isoorientin | H8914 | O802 | Pro 86 | H-donor | 1.75 | -9.93 |
| Rutin | O8890 | NG868 | His90 | H-donor | 1.99 | -7.712 |
| Isoquercitrin | O8895 | ND868 | His90 | H-donor | 2.49 | -12.12 |
| Isorhamnetin-3-O-glucoside | O8892 | OG5185 | Ser353 | H-donor | 2.67 | -10.72 |
| Myricetin | H8890 | OE2561 | Gln192 | H-donor | 2.58 | -13.69 |
| Quercetin | O8884 | ND868 | His90 | H-donor | 2.80 | -13.01 |
| Apigenin | O8882 | OH5736 | Tyr385 | H-donor | 2.67 | -12.12 |
| Kaempferol | No interaction |  |  |  |  |  |
| isorhamnetin | O8885 | OH5736 | Tyr385 | H-donor | 2.40 | -12.50 |
| Rhamnetin | O8885 | ND868 | His90 | H-donor | 2.48 | -13.09 |
|  | O8885 | OH5213 | Tyr355 | H-donor | 2.21 |  |

Reference

1 Saboo, S., Tapadiya, R., Khadabadi, S. & Deokate, U. In vitro antioxidant activity and total phenolic, flavonoid contents of the crude extracts of *Pterospermum acerifolium* wild leaves (Sterculiaceae). *J. Chem. Pharm. Res.* **2**, 417-423 (2010).

2 Kiranmai, M., Kumar, C. M. & Mohammed, I. Comparison of total flavanoid content of *Azadirachta indica* root bark extracts prepared by different methods of extraction. *Res. J. Pharm, Biol. Chem. Sci.* **2**, 254-261 (2011).

3 Justino, A. B. *et al.* *Annona muricata* Linn. leaf as a source of antioxidant compounds with in vitro antidiabetic and inhibitory potential against *α*-amylase, *α*-glucosidase, lipase, non-enzymatic glycation and lipid peroxidation. *Biomed. Pharmacother.* **100**, 83-92, doi:https://doi.org/10.1016/j.biopha.2018.01.172 (2018).

4 Ibrahim, R. M. *et al.* Metabolites profiling, in-vitro and molecular docking studies of five legume seeds for Alzheimer’s disease. *Sci. Rep.* **14**, 19637, doi:https://doi.org/10.1038/s41598-024-68743-7 (2024).

5 Mabry, T., Markham, K. & Thomas, M. The Systematic Identification of Flavonoids, Berlin-Heidelberg-New York: Springer Verlag. (1970).

6 George, A. *et al.* Anti-inflammatory effects of *Polygonum minus* (Huds) extract (Lineminus™) in in-vitro enzyme assays and carrageenan induced paw edema. *BMC Complement Altern. Med.* **14**, 1-7, doi:https://doi.org/10.1186/1472-6882-14-355 (2014).

7 Costamagna, M. S. *et al.* Polyphenols rich fraction from *Geoffroea decortican*s fruits flour affects key enzymes involved in metabolic syndrome, oxidative stress and inflammatory process. *Food Chem.* **190**, 392-402, doi:https://doi.org/10.1016/j.foodchem.2015.05.068 (2016).

8 Rahman, M. N. *et al.* X-ray Crystal Structure of Human Heme Oxygenase-1 with (2 R, 4 S)-2-[2-(4-Chlorophenyl) ethyl]-2-[(1 H-imidazol-1-yl) methyl]-4 [((5-trifluoromethylpyridin-2-yl) thio) methyl]-1, 3-dioxolane: A Novel, Inducible Binding Mode. *J. Med. Chem,* **52**, 4946-4950, doi:https://doi.org/10.1021/jm900434f (2009).

9 Gilbert, N. C. *et al.* Conversion of human 5-lipoxygenase to a 15-lipoxygenase by a point mutation to mimic phosphorylation at Serine-663. *FASEB J,* **26**, 3222, doi:https://doi.org/10.1096/fj.12-205286 (2012).

10 Jnoff, E. *et al.* Binding mode and structure–activity relationships around direct inhibitors of the Nrf2–Keap1 complex. *ChemMedChem* **9**, 699-705, doi:https://doi.org/10.1002/cmdc.201300525 (2014).

11 Kurumbail, R. G. *et al.* Structural basis for selective inhibition of cyclooxygenase-2 by anti-inflammatory agents. *Nature* **384**, 644-648, doi:https://doi.org/10.1038/384644a0 (1996).

12 Pettersen, E. F. *et al.* UCSF Chimera—a visualization system for exploratory research and analysis. *J. Comput. Chem,* **25**, 1605-1612, doi:https://doi.org/10.1002/jcc.20084 (2004).

13 Li, H., Robertson, A. D. & Jensen, J. H. Very fast empirical prediction and rationalization of protein pKa values. *Proteins: Structure, Function, and Bioinformatics* **61**, 704-721, doi:https://doi.org/10.1002/prot.20660 (2005).

14 Halford, B. Reflections on CHEMDRAW. *Chem. Eng. News* **92**, 26-27, doi:http://dx.doi.org/10.1021/cen-09233-scitech1 (2014).

15 Hanwell, M. D. *et al.* Avogadro: an advanced semantic chemical editor, visualization, and analysis platform. *J. Cheminform,* **4**, 1-17, doi:https://doi.org/10.1186/1758-2946-4-17 (2012).

16 Trott, O. & Olson, A. J. AutoDock Vina: improving the speed and accuracy of docking with a new scoring function, efficient optimization, and multithreading. *J. Comput. Chem,* **31**, 455-461, doi:https://doi.org/10.1002/jcc.21334 (2010).

17 Bikadi, Z. & Hazai, E. Application of the PM6 semi-empirical method to modeling proteins enhances docking accuracy of AutoDock. *J. Cheminform,* **1**, 1-16, doi:https://doi.org/10.1186/1758-2946-1-15 (2009).

18 Huey, R. & Morris, G. M. Using autodock with autodocktools: a tutorial. *The Scripps Research Institute Molecular Graphics Laboratory. California, USA* (2006).

19 Morris, G. M. *et al.* Automated docking using a Lamarckian genetic algorithm and an empirical binding free energy function. *J. Comput. Chem,* **19**, 1639-1662, doi:https://doi.org/10.1002/(SICI)1096-987X(19981115)19:14<1639::AID-JCC10>3.0.CO;2-B (1998).

20 Hospital, A., Goñi, J. R., Orozco, M. & Gelpí, J. L. Molecular dynamics simulations: advances and applications. *Adv. Appl. Bioinforma. Chem.*, 37-47, doi:https://doi.org/10.2147/AABC.S70333 (2015).

21 Lee, T.-S. *et al.* GPU-accelerated molecular dynamics and free energy methods in Amber18: performance enhancements and new features. *J. Chem. Inf. Model.* **58**, 2043-2050, doi:https://doi.org/10.1021/acs.jcim.8b00462 (2018).

22 Wang, J., Wang, W., Kollman, P. A. & Case, D. A. Automatic atom type and bond type perception in molecular mechanical calculations. *J. Mol. Graph. Model.* **25**, 247-260, doi:https://doi.org/10.1016/j.jmgm.2005.12.005 (2006).

23 Berendsen, H. J., Postma, J. v., Van Gunsteren, W. F., DiNola, A. & Haak, J. R. Molecular dynamics with coupling to an external bath. *J. Chem. Phys.* **81**, 3684-3690, doi:https://doi.org/10.1063/1.448118 (1984).

24 Roe, D. R. & Cheatham III, T. E. PTRAJ and CPPTRAJ: software for processing and analysis of molecular dynamics trajectory data. *J. Chem. Theory Comput.* **9**, 3084-3095, doi:https://doi.org/10.1021/ct400341p (2013).

25 Seifert, E. OriginPro 9.1: scientific data analysis and graphing software-software review. *J. Chem. Inf. Model.* **54**, 1552, doi:https://doi.org/10.1021/ci500161d (2014).

26 Ylilauri, M. & Pentikäinen, O. T. MMGBSA as a tool to understand the binding affinities of filamin–peptide interactions. *J. Chem. Inf. Model.* **53**, 2626-2633, doi:https://doi.org/10.1021/ci4002475 (2013).

27 Hou, T., Wang, J., Li, Y. & Wang, W. Assessing the performance of the MM/PBSA and MM/GBSA methods. 1. The accuracy of binding free energy calculations based on molecular dynamics simulations. *J. Chem. Inf. Model.* **51**, 69-82, doi:https://doi.org/10.1021/ci100275a (2011).

28 Greenidge, P. A., Kramer, C., Mozziconacci, J.-C. & Wolf, R. M. MM/GBSA binding energy prediction on the PDBbind data set: successes, failures, and directions for further improvement. *J. Chem. Inf. Model.* **53**, 201-209, doi:https://doi.org/10.1021/ci300425v (2013).

29 Pehar, V., Oršolić, D. & Stepanić, V. in *17. Ružičkini dani" Danas znanost-sutra industrija".* 112-123.

30 Lipinski, C. A., Lombardo, F., Dominy, B. W. & Feeney, P. J. Experimental and computational approaches to estimate solubility and permeability in drug discovery and development settings. *Adv. Drug Deliv. Rev.* **23**, 3-25, doi:https://doi.org/10.1016/S0169-409X(96)00423-1 (1997).

31 Daina, A., Michielin, O. & Zoete, V. SwissADME: a free web tool to evaluate pharmacokinetics, drug-likeness and medicinal chemistry friendliness of small molecules. *Sci. Rep.* **7**, 42717, doi:https://doi.org/10.1038/srep42717 (2017).

32 Mo, E., Ebedy, Y. A., Ibrahim, M. A., Farroh, K. Y. & Hassanen, E. I. Newly synthesized chitosan-nanoparticles attenuate carbendazim hepatorenal toxicity in rats via activation of Nrf2/HO1 signalling pathway. *Sci. Rep.* **12**, 9986, doi:https://doi.org/10.1038/s41598-022-13960-1 (2022).

33 Ebedy, Y. A., Hassanen, E. I., Hussien, A. M., Ibrahim, M. A. & Elshazly, M. Neurobehavioral toxicity induced by carbendazim in rats and the role of iNOS, Cox-2, and NF-κB signalling pathway. *Neurochem. Res.* **47**, 1956-1971, doi:https://doi.org/10.1007/s11064-022-03581-5 (2022).

34 Hassanen, E. I. *et al.* Potential mechanisms of imidacloprid-induced neurotoxicity in adult rats with attempts on protection using Origanum majorana L. oil/extract: In Vivo and In Silico Studies. *ACS omega* **8**, 18491-18508, doi:https://doi.org/10.1021/acsomega.2c08295 (2023).

35 Hassanen, E. I. *et al.* Mechanistic approach on the pulmonary oxido-inflammatory stress induced by cobalt ferrite nanoparticles in rats. *Biol. Trace Elem. Res.* **202**, 765-777, doi:https://doi.org/10.1007/s12011-023-03700-5 (2024).
